# Rosuvastatin Enhances VSV-G Lentiviral Transduction of NK Cells via Upregulation of the Low-Density Lipoprotein Receptor

**DOI:** 10.1016/j.omtm.2020.03.017

**Published:** 2020-03-29

**Authors:** Ying Gong, Roel G.J. Klein Wolterink, Ian Janssen, Arjan J. Groot, Gerard M.J. Bos, Wilfred T.V. Germeraad

**Affiliations:** 1 Department of Internal Medicine, Division of Hematology, Maastricht University Medical Center+, Maastricht, the Netherlands; 2 GROW - School for Oncology and Developmental Biology, Maastricht University, Maastricht, the Netherlands; 3 Department of Radiology, Maastricht University, Maastricht, the Netherlands; 4 CiMaas BV, Maastricht, the Netherlands

**Keywords:** natural killer cells, VSV-G, lentiviral transduction, statins, rosuvastatin, GGPP, LDLR

## Abstract

Adoptive natural killer (NK) cell therapy is attaining promising clinical outcomes in recent years, but improvements are needed. Genetic modification of NK cells with a tumor antigen-specific receptor on their surface coupled to intracellular signaling domains may lead to enhanced cytotoxicity against malignant cells. One of the most common approaches is by lentivirus-mediated transduction. However, NK cells are difficult to transduce and various methods have been attempted with different success rates. Because the low-density lipoprotein-receptor (LDLR) is the receptor of vesicular stomatitis virus (VSV) and is expressed only at low levels on NK cells, we tested the potential of 5 statins and 5 non-statin compounds to increase the LDLR expression, thereby facilitating viral transduction. We found that the transduction efficiency of VSV-G pseudotyped lentivirus is augmented by statins that induced higher LDLR expression. In both NK-92 cells and primary NK cells, the transduction efficiency increased after treatment with statins. Furthermore, statins have been reported to suppress NK cell cytotoxicity; however, we showed that this can be completely reversed by adding geranylgeranyl-pyrophosphate (GGPP). Among the statins tested, we found that the combination of rosuvastatin with GGPP most potently improved viral transduction without affecting the cytotoxic properties of the NK cells.

## Graphical Abstract

**Figure:**
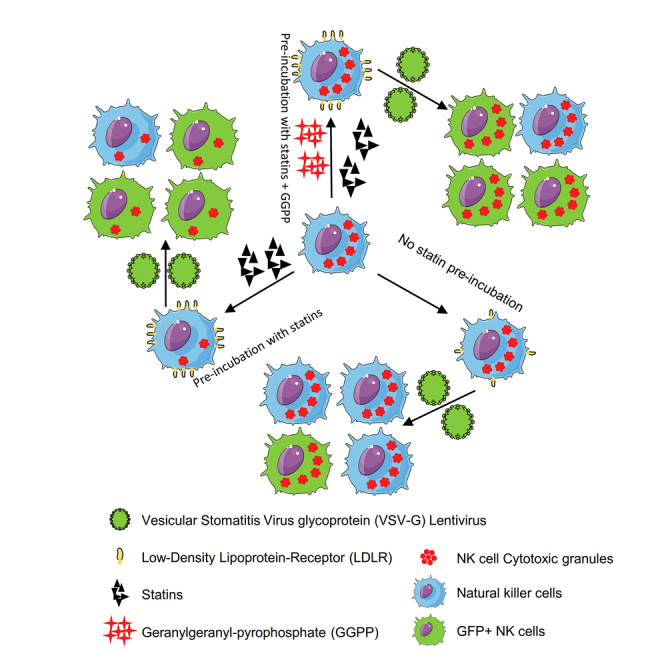

## Teaser

Natural killer (NK) cells are known to be difficult to transduce, and various methods have been attempted with different success rates. Gong and colleagues found that the combination of rosuvastatin with GGPP most potently improved VSV-G lentivirus transduction via upregulating cell surface LDLR without affecting the cytotoxic properties of the NK cells.

## Introduction

Cancer immunotherapy, with its higher specificity and fewer side effects compared to traditional anti-cancer therapies, has become an important tumor therapeutic strategy.^1^ The main purpose is to break tolerance and revitalize the body’s immune system that has become insensitive for advanced malignancies. The most prominent advantage of immunotherapy is the potential ability to eradicate distant metastases leading to a possible cure in a percentage of patients. A sophisticated form of immunotherapy is cellular therapy that includes dendritic cell vaccination, adoptive therapy of tumor infiltrate lymphocytes (TIL), tumor-specific T cell receptor T cells (TCR-T), and natural killer (NK) cells, as well as chimeric antigen receptor (CAR)-T and CAR-NK.

NK cells are innate immune cells having a surveillance function to eradicate virally infected cells or malignant cells. NK cells have several cytotoxic components leading to destruction of target cells. Perforin and granzymes incorporate into granules in the cytoplasm once NK cells are educated. After NK cells have recognized and bound target cells, the granules merge with the cell membrane, and they secrete their content to mediate the killing process of the target cell by inducing apoptosis along the caspase pathways. One of the main mechanisms of NK cells to become active and cytotoxic is the concept of “missing self.” NK cells can recognize cells that are missing major histocompatibility complex (MHC) class I and thereby become activated. Moreover, NK cells can directly recognize tumor cells independent of MHC presentation like T cells.^2^ The other mechanisms by which NK cells kill, include the death receptors CD178 (Fas Ligand [FasL]) and tumor necrosis factor-related apoptosis-inducing ligand (TRAIL), also leading to apoptosis, but are different in terms of timing.^3^

Adoptive transfer of mature alloreactive NK cells was shown to be effective in the treatment of patients with acute myeloid leukemia (AML) and to prevent relapse.^4^ In contrast to the percentage of T cells, NK cells comprise a relatively small population and are not persistent as long as T cells *in vivo*.

With the development of genetic modification methods and promising clinical outcome of gene engineered T cells, NK cells could be great effector cells once armed with a specific antigen ligand or antibody. Building on the first clinical successes and subsequent application of the CD19 CAR-T for B cell hematological malignancies, there are increasing CAR-T or, more recently, CAR-NK clinical trials in progress to treat cancer patients.^5^^,^^6^

Generating CAR-T or CAR-NK cells consists of a genetic modification of cells resulting in surface expression of the antigen binding part of an antibody coupled to intracellular T cell or NK cell signaling molecules. This structure endows the T cell or NK cell to directly recognize the native tumor antigen,^7^ resulting in stronger cell activation and enhanced cytotoxicity.

Despite the sensational clinical results with CAR-T cells, including their long *in vivo* persistence, it comes with the potential of various side effects; especially a cytokine release storm and neurotoxicity may cause dramatic outcomes and even death.^8^ In this concept, NK cells with their short lifespan and high killing capacity could form an alternative and effective cell therapy.^4^ Furthermore, combining a best-of-both-worlds concept, a CAR-NK cell can be generated.

Genetic modification to generate CAR-NK cells is aimed to improve their killing ability and tumor antigen targeting capacity. However, high efficiency of transfection or transduction of NK cells remains a big challenge. Retroviruses or lentiviruses are the transfer methods of choice to obtain permanent integration of the transgene with high transduction efficiencies. Numerous reagents have been used to enhance viral transduction. Protamine sulfate or polymers (dextran or polybrene) can eliminate the electronic charge on the cell membranes.^9^ Cyclosporine A^10^ and rapamycin relieve distinct lentiviral restriction blocks in hematopoietic stem and progenitor cells.^11^ Tolga et al.^12^ reported that inhibition of intracellular antiviral defense mechanisms augments lentiviral transduction of human NK cells. Vectofusin-1^13^ and prostaglandin E2^9^ and dextran^11^ have been reported to enhance lentiviral vector transduction of human hematopoietic stem cells (HPSCs), T lymphocytes,^14^ and primary NK cells,^15^ respectively, without further mechanistic description.

Vesicular stomatitis virus G protein (VSV-G) can be used as an envelope protein on the lentiviral particles,^16^ and the low density lipids (LDL) receptor and its family members serve as the cellular VSV receptors in human primary lymphocytes.^17^ Upregulation of the LDL receptor on lymphocytes may improve the VSV-G lentiviral transduction.^18^ Interestingly, various groups have shown that the expression levels of LDLR in human B and T lymphocytes can be increased using antibodies, cytokines, and estrogen receptor modulators.^18^^,^^19^ Clinicians used statins as anti-hyperlipidemia drugs because they will upregulate the LDL receptor on endothelial cells thereby increasing lipid removal from the blood. However, in NK cells, the impact of LDLR expression and its modulators has not been investigated. Therefore, we investigated which compounds influence the LDLR expression levels on NK cells and how LDLR expression levels improve lentiviral transduction efficiency of NK cells while NK cells ultimately maintain their cytotoxic capacity.

## Results

### Statins Enhance LDLR Expression Levels in the NK-92 Cell Line

Given that LDLR expression levels in human B and T lymphocytes can be influenced using compounds compatible with *in vitro* culture, we first asked what drugs influence LDLR expression levels in human NK cells. For screening purposes, we made use of the human NK cell line NK-92. This cell line shares important features with primary NK cells: it recognizes viruses and tumor cells, has cytotoxic capabilities, and produces characteristic NK cell cytokines.^20^ Based on previous publications, we tested compounds that have been reported to enhance NK cell transduction (interleukin-21 [IL-21]^21^ and dextran^15^), enhance lentiviral transduction in hematopoietic stem cells and T lymphocytes (vectofusin-1^14^ and prostaglandin E2^22^), and promote NK cell proliferation (ascorbic acid).^23^ Furthermore, we tested statins (high-mobility group-coenzyme A [HMG-CoA] reductase inhibitors) that are clinically used as lipid-lowering medication^24^ and that have been reported to directly increase *LDLR* mRNA in human mononuclear cells.^25^ Here, we examined the effects of three lipophilic statins (atorvastatin, fluvastatin, and simvastatin) and two hydrophilic statins (pravastatin and rosuvastatin).

**Figure 1:**
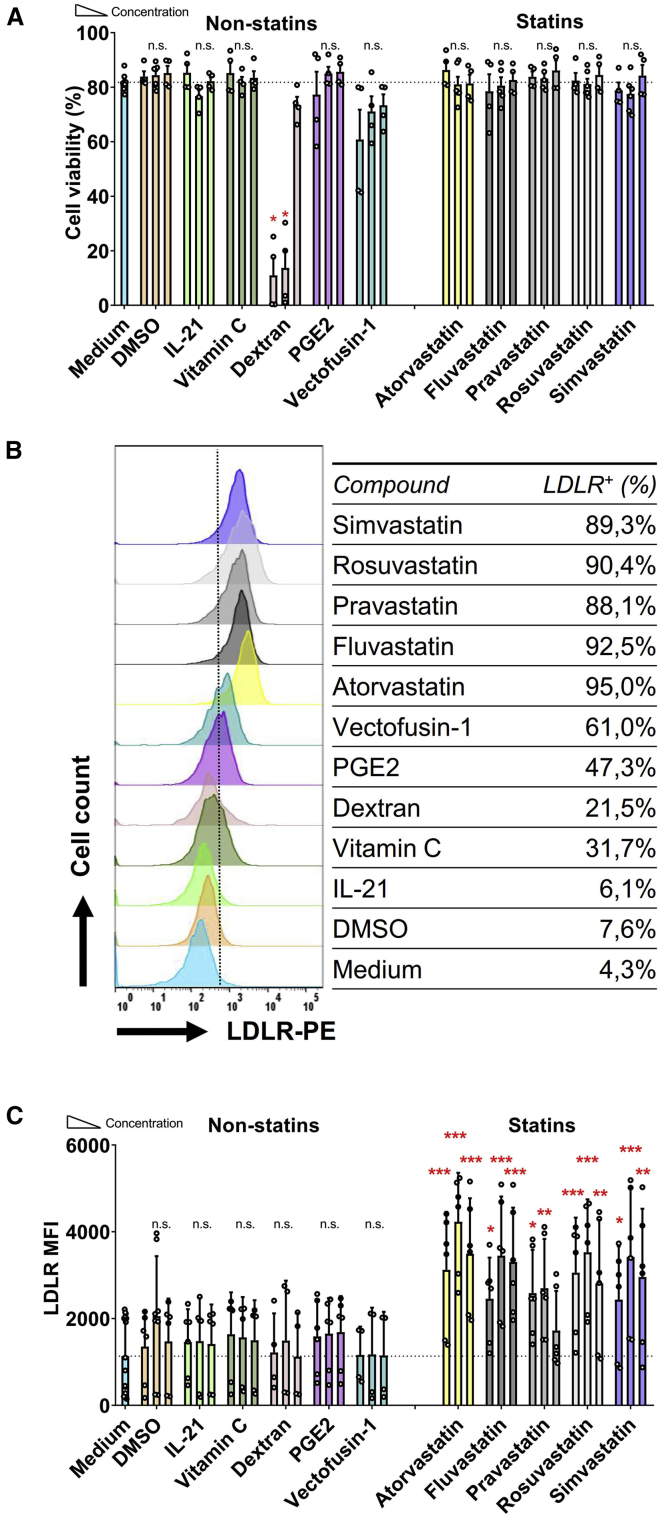
LDL-Receptor Upregulated by Statins on NK-92 Cells (A) Viability of NK-92 cells after 36 h of co-culture with different concentrations of compounds in MEM medium with 100 U/mL IL-2. Dead cells were stained by the fixable Aqua V500. NK-92 cells were seeded at 0.1 × 106/mL in 96-well plates, 200 μL in each well. IL-21 was added at 20 ng/mL, 5 ng/mL, and 0.5 ng/mL. Vitamin C was used at concentrations of 500 μg/mL, 50 μg/mL, and 5 μg/mL. Dextran was used at 80 μg/mL, 8 μg/mL, and 0.8 μg/mL. PGE2 was used at 100 μM, 10 μM, and 1 μM. Vectofusin-1 was used at 50 μg/mL, 5 μg/mL, and 0.5 μg/mL. All the statins were used at 20 μM, 5 μM, and 0.5 μM. Concentration height is from left to right, indicated with the triangle. DMSO was used as solvent for the statins and was taken along as negative control (B) LDLR expression level of NK-92 cells after 36 h co-culture with the compounds are expressed as median fluorescence intensity (MFI). Overlay histogram of LDLR expression on NK-92 cells under every middle concentration of all compounds are displayed. The table indicates the fraction of LDLR-positive cells. (C) LDLR levels were upregulated by statins, but not by non-statin compounds. Data are shown as mean ± SD; Pooled data from 4 independent experiments performed at different times. Data analysis was performed by a two-way ANOVA and Bonferroni post-tests in comparison to medium. For cell viability, Mann-Whitney U test was used to compare with medium group.

Previously, Hillyard et al.^26^ had demonstrated that 10 μM statin is detrimental for the NK cell membrane raft, which is a key functional component for NK cell cytotoxicity. The same statins concentration was also shown by Poggi et al.,^27^ who reported that 10 μM fluvastatin was able to decrease the activation markers on NK cells. Initially, we tested three different concentrations of statins: 0.5 μM, 5 μM, and 20 μM. Higher doses of 50 μM resulted in a 50% reduction in viability after 36 h incubation and were therefore not used (data not shown). Culture of NK-92 cells with the various statins at these concentrations did not have a significant negative impact on cell viability (Figure 1A). Similarly, most non-statin compounds, except dextran, did not negatively impact NK-92 cell viability, even at the highest concentrations tested (Figure 1A).

Flow cytometric analysis showed that all statins tested increased the LDLR protein expression at least 3-fold, while none of the other compounds significantly upregulated LDLR expression levels (Figures 1B and 1C). LDLR expression levels were not strictly dose-dependent: we observed that LDLR expression levels were generally highest following culture in 5 μM of the respective statins (Figure 1C), without a negative impact on cell viability (Figure 1A). Therefore, we determined 5 μM to be the optimal concentration to augment the expression of LDLR on NK-92 cells. Statins at higher concentrations inhibit the viability of NK cells, whereas at a low 0.5 μM LDLR expression was not highly induced. The major impact of the statins is apparent within 12 h (Figure S1A) and the induction of LDLR expression on NK-92 cells following statin stimulation is time-dependent. Moreover, addition of statins also impacted on cell viability, as 48 h after statin administration, the NK cells that normally grow in clumps loosened, resulting in a gradual decrease in overall viability (Figure S1B), while LDLR expression levels did not increase further (Figure S1A). Therefore, we determined 5 μM to be the optimal concentration and 36 h incubation for statins to upregulate LDLR expression in NK-92 cells, since LDLR expression was highest at this concentration with no negative impact on cell viability (Figures S1B and S1C).

### Statins Enhance the Viral Transduction Efficiency of NK-92 Cells

**Figure 2:**
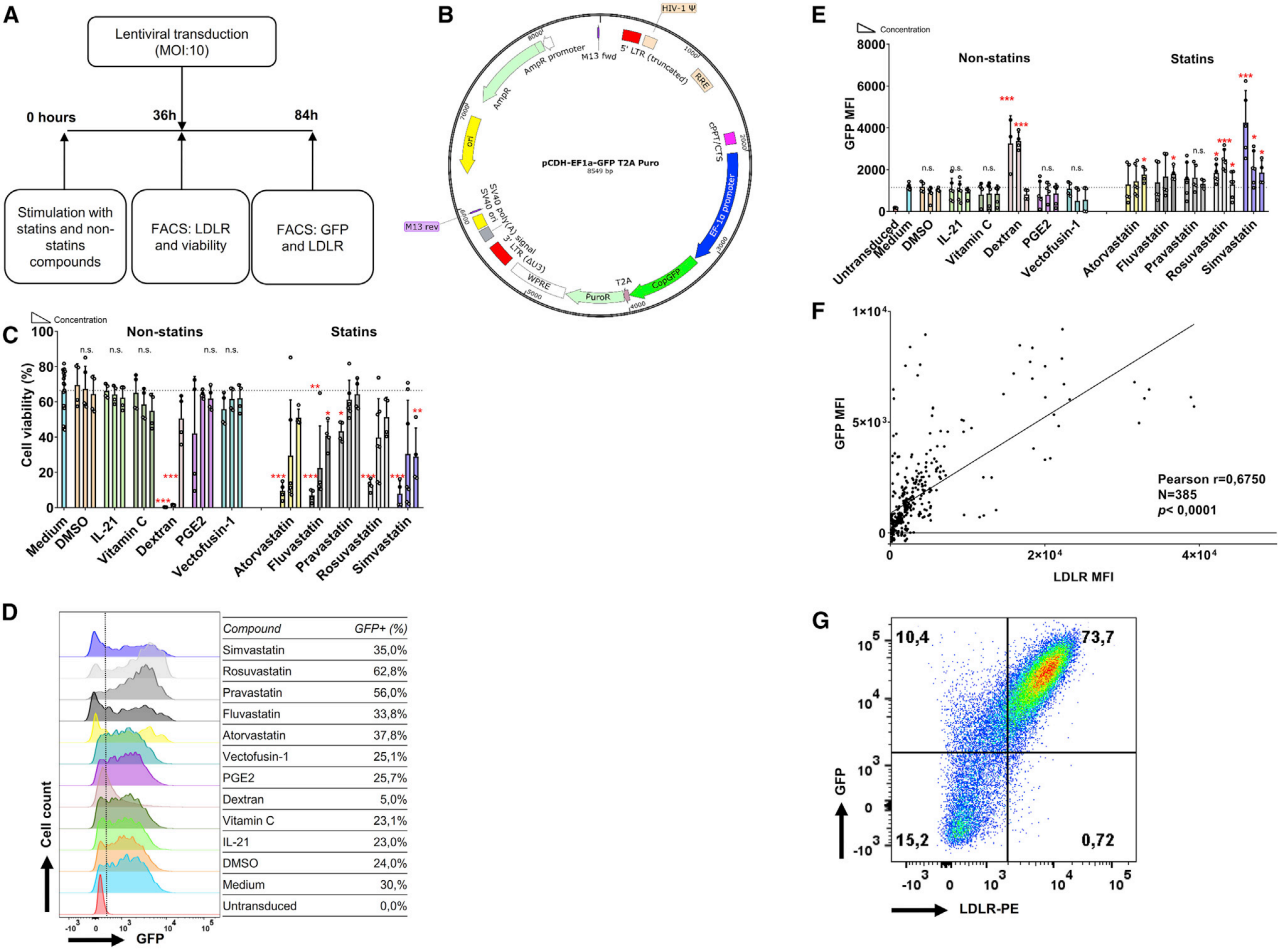
NK-92 Cell Transduction Efficiency Is Enhanced by Statins (A) Flow chart of procedures using NK-92 cells for transduction with or without compounds. (B) Map of 3rd generation lentiviral vector pCDH-EF1a-GFP-puro. (C) Viability of NK-92 cells 48 h after viral transduction, following culture in the presence of the different compounds at the indicated concentrations. After co-culturing for 36 h, medium was exchanged in each well. Dead cells were detected by fixable Aqua V500. (D) Flow cytometric overlay histograms of GFP expression in NK-92 cells cultured with the middle concentration of each compound. VSV-G lentivirus was added at the MOI of 10:1 in the presence of 10 μg/mL protamine sulfate for transduction. (E) NK cell transduction efficiency was determined by GFP expression in living NK-92 cells. (F) Pearson correlation analysis between LDLR MFI before transduction and 48 h after transduction of NK-92 cells. (G) One representative example of LDLR co-expression with GFP on NK-92 48 h after viral transduction was showed. Data shown as mean ± SD. Pooled data from n = 4 independent experiments performed at different times.

Next, we determined whether the statin-induced increase of cell surface LDLR expression levels in NK-92 cells led to enhanced VSV-G lentiviral transduction efficiency. Thus, we treated cultured NK-92 cells with statins for 36 h, followed by lentiviral transduction with a GFP-encoding vector and analysis after 48 h (Figures 2A and 2B). We used flow cytometric analysis of the fraction of GFP-positive cells and their mean fluorescence index (MFI) to determine the lentiviral transduction efficiency. As reported by other groups,^7^^,^^21^ lentiviral transduction of NK-92 cells has a mild negative impact on cell viability (Figure 2C). However, in combination with lentiviral transduction, most statins unexpectedly had a profound impact on cell viability. Notably, this effect was less pronounced in the cultures treated with the hydrophilic statins pravastatin and rosuvastatin compared with the lipophilic statins (Figure 2C). Importantly, atorvastatin, rosuvastatin, and simvastatin significantly increased the fraction of GFP-expressing cells and the GFP expression levels 1.5- to 2.5-fold, indicating improved transduction efficiency (Figures 2D and 2E). In the non-statins group, only dextran promoted transduction efficiency at the high expense of cell viability. Interestingly, higher doses of statins did not further enhance GFP expression levels after lentiviral transduction (Figure 2E), while the observed negative effects of statins on cell viability of lentivirus transduced NK-92 cells were dose-dependent (Figure 2C). Pooled analysis of the effect of LDLR expression levels on transduction efficiency showed that higher LDLR expression analysis correlated with higher transduction efficiency (Pearson correlation coefficient r = 0.6750, p < 0.0001) (Figures 2F and 2G). In addition, we observed that higher virus titers resulted in higher GFP expression levels, but also led to more cell death (Figure S2). Therefore, we determined a MOI of 10 to be the optimal virus concentration for the transduction of NK-92 cells. Statins enhanced lentiviral transduction efficiency of NK-92 cells around 2-fold when treated with atorvastatin, rosuvastatin, or simvastatin. While lentiviral transduction in the presence of statins has a negative impact on cell viability, this effect is dose-dependent, and a 5 μM dose of statins can be used without major negative effects on cell viability.

### Statin-Induced Reduction in NK Cell Cytotoxicity Is Reversed by GGPP

**Figure 3:**
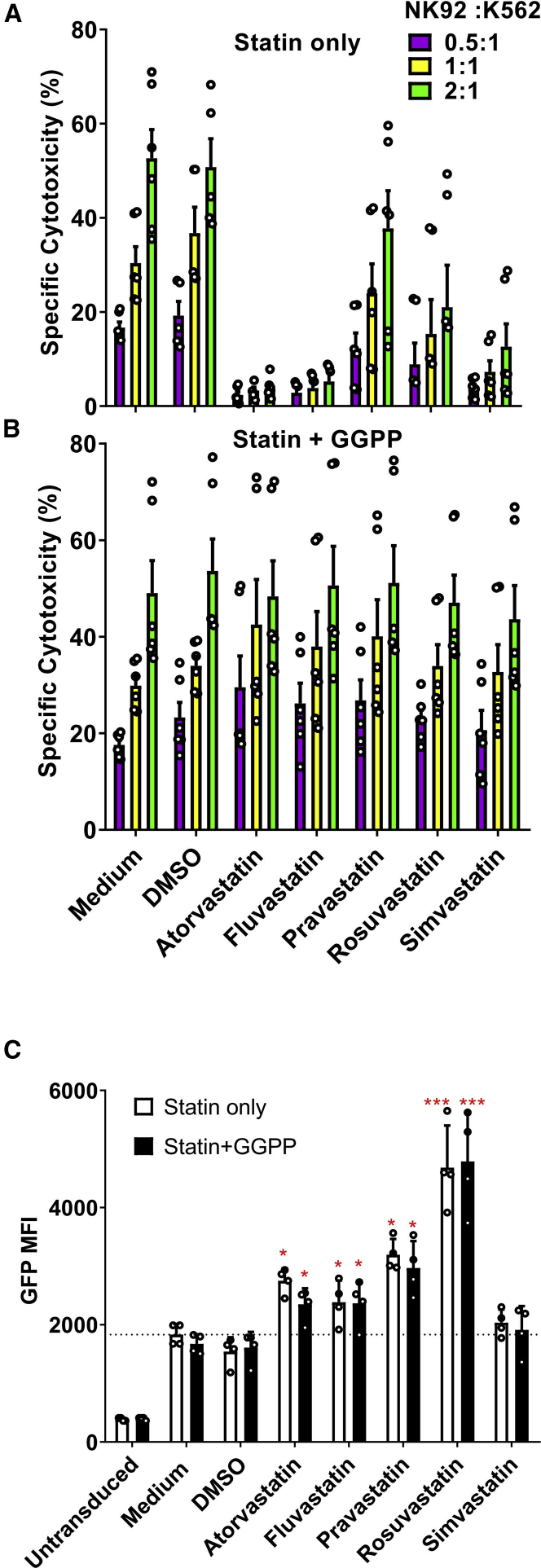
Statins Inhibited NK-92 Cell Cytotoxicity, which Could Reversed by GGPP without Influencing GFP Expression (A) NK-92 cell cytotoxicity was suppressed by 5 μM lipophilic statins after 36 h incubation. However, NK-92 cells’ cytotoxicity was not changed by pravastatin and not 100% restrained by rosuvastatin. NK-92 cells were counted after 36 h exposure to statins and mixed in specified ratios of effector cells to target cells (E/T ratio) in 96-well plates. K562 target cells were seeded at 20,000 cells per well. The cytotoxicity assay was performed during 4 h. Cytotoxicity was quantified as percentage dead target cells using flow cytometry. (B) The inhibition of statins on NK-92 cell cytotoxicity could be reversed by 10 μM GGPP. Data shown is 1 representative experiment out of 3 independent assays. (C) The statin induced enhancement of NK-92 cells transduction efficiency was not altered by GGPP. NK-92 cells were incubated with statins in the presence of GGPP or not during 36 h. Then, culture medium was refreshed and VSV-G at MOI of 10:1 was added. Data were showed as mean ± SD and derived from n = 4 independent experiments performed at different times. Data analysis was performed by pair signed-rank test between statins only group and statins plus GGPP group.

NK cell immunotherapy builds on the intrinsic cytotoxic capacity of the cells. For NK cells, it has previously been demonstrated that statins can inhibit their cytotoxic capacity.^26^^,^^28^ Indeed, using the NK-92 cells, we observed almost complete inhibition of cytotoxicity against the K562 chronic myelogenous leukemia (CML) cell line 48 h after lentiviral transduction in the presence of atorvastatin, fluvastatin, or simvastatin, while cytotoxicity was slightly decreased in the presence of rosuvastatin (Figure 3A). However, pravastatin did not alter the cytotoxic capacity of NK-92 cells on K562 cells. Interestingly, geranylgeranyl-pyrophosphate (GGPP), a key molecule in biosynthesis downstream pathways of HMG-CoA reductase,^29^ could completely reverse the suppression statin-induced NK cell cytotoxicity (Figure 3B), while maintaining the statin-induced increase in lentiviral transduction (Figure 3C). We also tested whether the cytokine IL-2, that is commonly used to activate NK cells and NK cell lines, could reverse the negative effects of statins on NK cell cytotoxicity, as IL-2 was previously shown to be sufficient to overcome the negative impact of statins on cytotoxicity.^30^ However, in NK-92 cells, a high dose of IL-2 (1,000 U/mL) alone was not sufficient to restore their cytotoxic capacity (Figure S3).

To unravel what NK cell cytotoxic pathway was impacted by statins, we analyzed expression of the degranulation marker CD107a, granzyme B, apoptosis-inducing FAS ligand and interferon-γ (IFN-γ) secretion (Figure S4). Indeed, we observed downregulation of CD107a and granzyme B levels in statin-treated NK-92 cells compared to control cells (Figures S4A and S4B). Importantly, these effects were reversed by GGPP, while GGPP did not influence any of the other analyzed cytotoxicity pathways. Thus, statins can importantly reduce NK-92 cell cytotoxicity, but GGPP can fully reverse this effect completely (Figure 3C).

### Statins Upregulate LDLR Expression and Enhance the Viral Transduction Efficiency of Primary Human NK Cells

**Figure 4:**
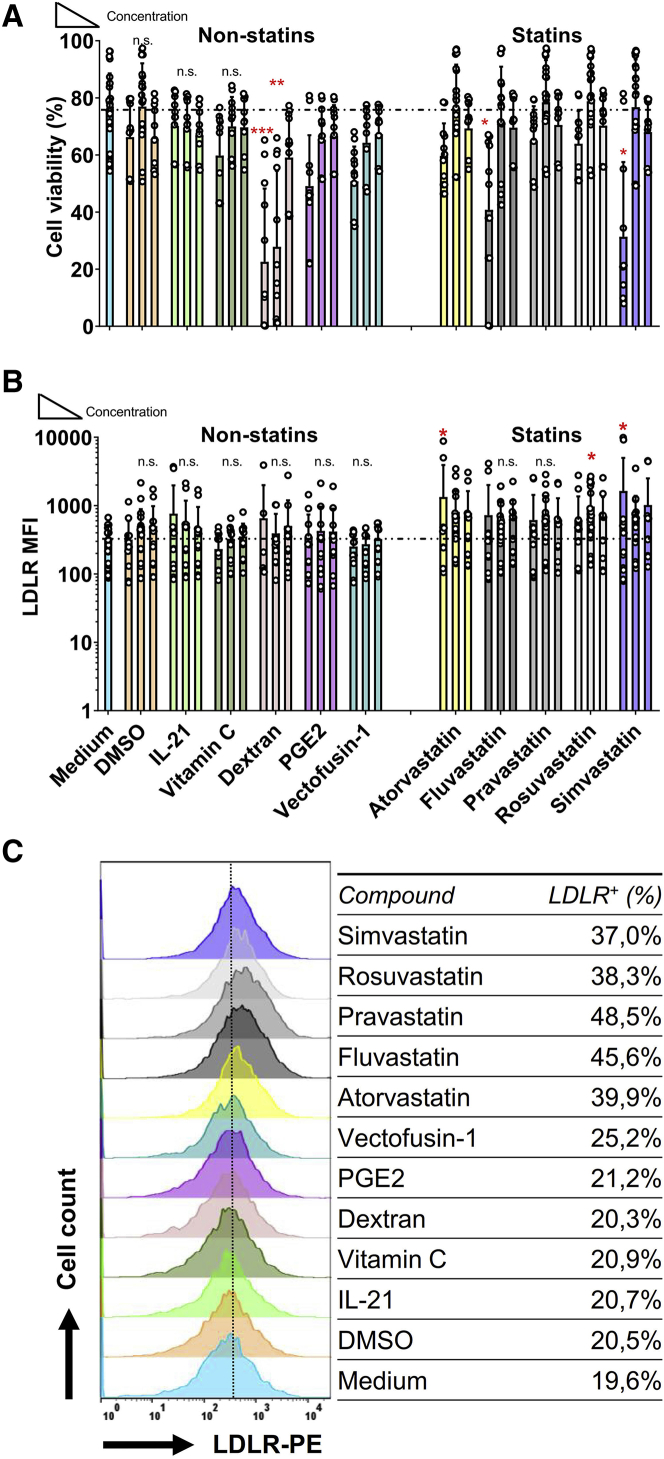
LDL-Receptor Upregulation by Statins on Human Primary NK Cells (A) Viability of NK cells after 36 h stimulation with different compounds as previous described. Human primary NK cells were co-cultured with compounds for 36 h at a density of 0.1 × 10^6^/mL. NK cells were pre-activated overnight in RPMI-1640 with 1,000 U/mL IL-2. (B) The LDLR expression on primary NK cell was upregulated by rosuvastatin, however, in the non-statin groups no significant difference was found when compared to medium. (C) Overlay histogram of LDLR expression on NK cells stimulated 36 h with different compounds. Data are from n = 6 independent experiments performed at different times. Data analysis was performed by a two-way ANOVA and Bonferroni post-tests in comparison to medium. For cell viability, Mann-Whitney U test was used to compare with medium group.

While lymphoma-derived NK-92 cells have been used for clinical applications,^31^ human primary NK cells are a heterogeneous population of cells that in general shows better cytotoxic capacities.^32^ We checked freshly isolated human primary NK cells and found that the CD56^dim^ population expressed more LDLR than the CD56^bright^ population (Figures S5A and S5B). Therefore, we next tested whether statins can also be used to enhance lentiviral transduction via LDLR upregulation in primary human NK cells. First, we confirmed that statins (5 μM) and the non-statin compounds did not negatively impact NK cell viability (Figure 4A). The only exception was dextran, which as previously shown had a marked negative impact on NK-92 viability (Figure 1A). In the statin-treated groups, we observed a trend of LDLR upregulation, which was only significantly upregulated 2-fold in the rosuvastatin-treated NK cells (Figures 4B and 4C). Further analysis of LDLR expression showed that most likely only a subset of primary NK cells responds to statin treatment (Figure 4C).

**Figure 5:**
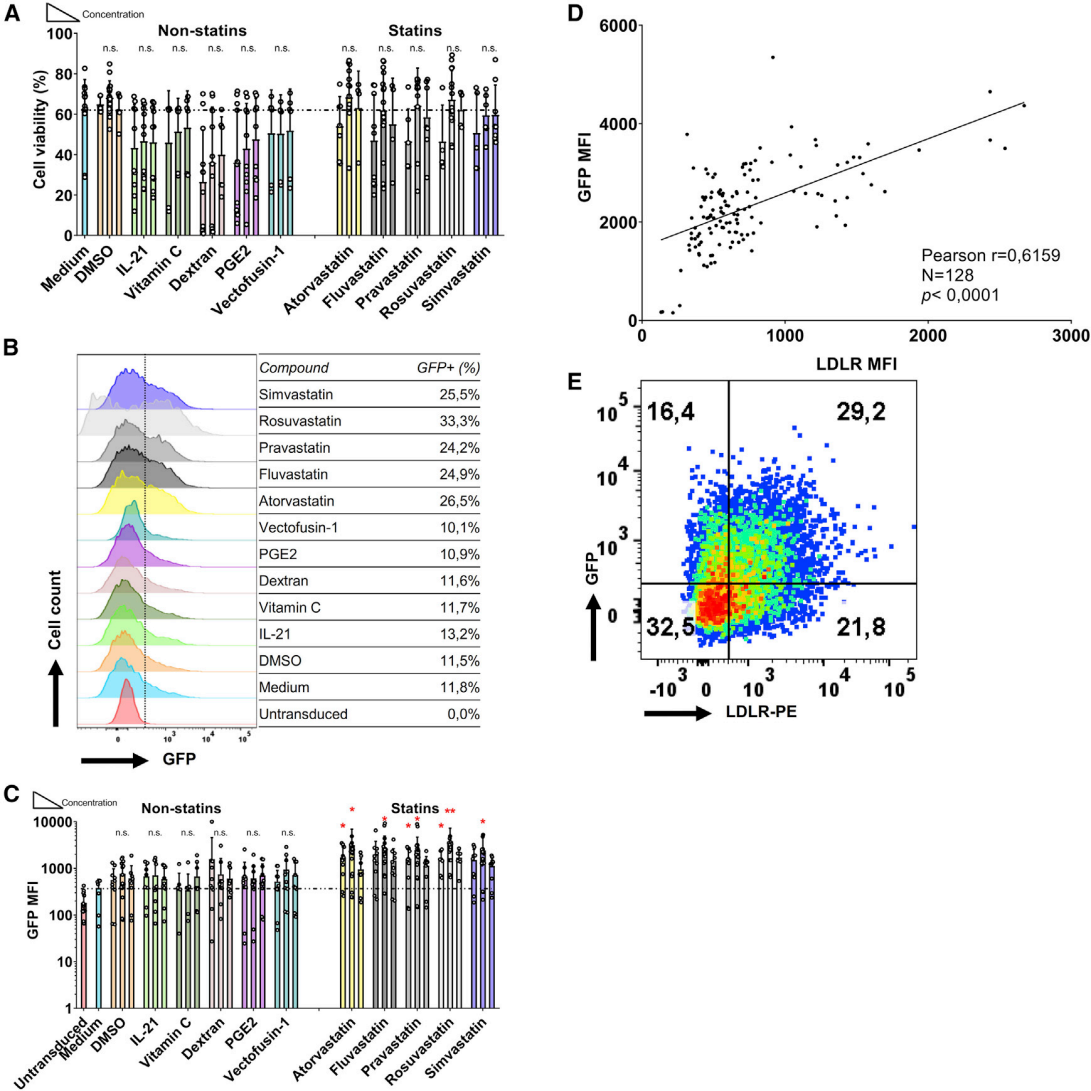
Lentiviral Transduction of Human Primary NK Cells (A) Viability of primary NK cells 48 h after transduction with different compounds at the indicated concentrations. (B) Flow cytometric overlay histogram of GFP expression in NK cells. VSV-G lentivirus was added at the MOI of 10:1 in the presence of 10 μg/mL protamine sulfate. (C) NK cell transduction efficiency was determined by GFP expression in living NK cells. GFP expression was higher after culture with rosuvastatin when compared with DMSO and medium group. (D) Pearson correlation analysis of LDLR MFI (before transduction) and GFP MFI (48 h after transduction) on NK cells. (E) One representative LDLR co-expression with GFP on NK after 48 h viral transduction. Data was showed as mean ± SD from n = 6 independent experiments performed at different times. Data analysis was performed by a two-way ANOVA and Bonferroni post-tests in comparison to medium. For cell viability, Mann-Whitney U test was used to compare with medium group.

We subsequently transduced primary NK cells with the GFP-expression lentiviral vector that we used before to transduce NK-92 cells (Figure 2A). In contrast to NK-92 cells, primary NK cells can be transduced in the presence of statins or non-statins without significant negative effects on cell viability. However, we did not observe significant differences in viability after treatment with statins (Figure 5A). Meanwhile, we observed a 2- to 3-fold increase in GFP expression levels in statin-treated NK cells compared to the non-statin control group (Figures 5B and 5C). 48 h after transduction, genomic DNA was extracted from the VSV-G transduced primary NK cells and PCR was used to confirm integration of transgene (Figure S6). Indeed, LDLR expression levels positively correlated with GFP expression levels after lentiviral transduction (Figures 5D and 5E; Pearson correlation coefficient r = 0.6159, p < 0.0001), demonstrating that rosuvastatin that induce LDLR expression can be used to enhance lentiviral transduction.

### Rosuvastatin plus GGPP Promotes Lentiviral Transduction through an Increase of LDLR Expression on the Surface of Primary NK Cells

**Figure 6:**
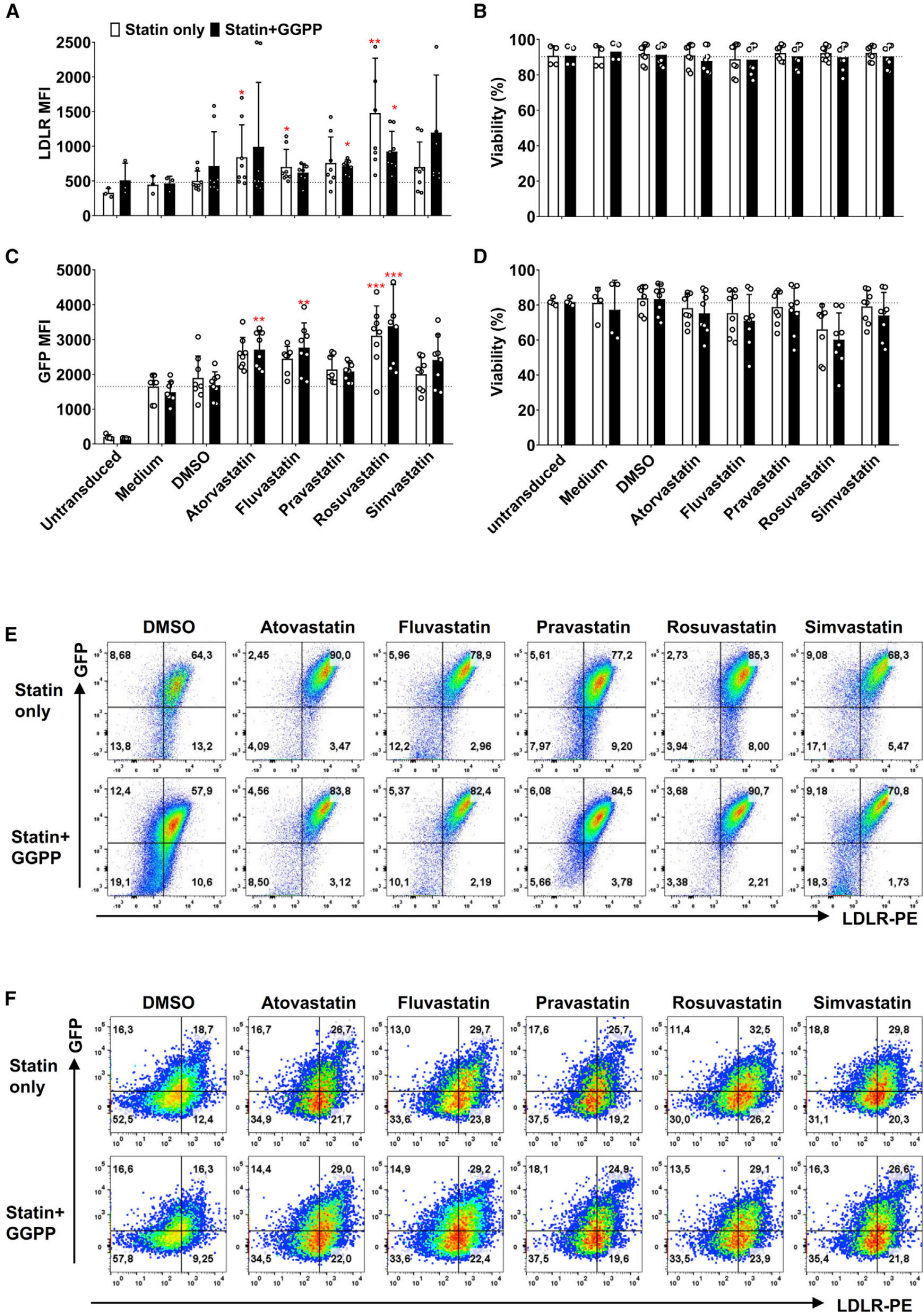
Statins with GGPP Enhance the Transduction Efficiency of NK-92 and Primary NK Cells (A) LDL-receptor expression on primary NK cells after 36 h stimulation with 5 μM statins, with or without 10 μM GGPP. Primary NK cells were co-cultured with 5 μM statins in the presence or absence of GGPP at a density of 0.1 × 106/mL (B) Viability of primary NK cells after 36 h cultured in the presence of 5 μM statins with or without 10 μM GGPP. (C) GFP expression in primary NK cells after lentiviral transduction. After statins incubation, the culture medium was refreshed and VSV-G added at MOI of 10:1. (D) Viability of primary NK cells after 48 h transduction. (E and F) Representative flow cytometry LDLR and GFP co-expression in NK-92 (E) and primary NK cells (F) after 48 h lentiviral transduction and culturing with statins with or without GGPP. Data is showed as mean ± SD from n = 4 independent experiments performed at different times. Data analysis was performed by a two-way ANOVA and Bonferroni post-tests in comparison to medium. For cell viability, Mann-Whitney U test was used to compare with medium group.

**Figure 7:**
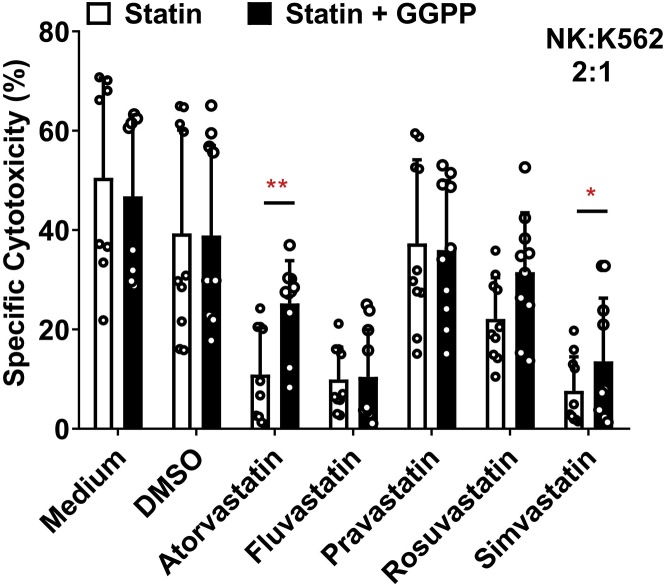
Cytotoxicity of Primary NK cells Is Inhibited by Statins, but Restored by GGPP Human primary NK cell cytotoxicity was suppressed by lipophilic statins. NK cell cytotoxicity was not changed by pravastatin and restrained by rosuvastatin. NK cells were incubated with statins in presence of GGPP or not. NK cells were seeded at the density of 0.1 × 10^6^/mL in 96-well plates with or without statins. After removing the supernatant, NK cells were added in a 2:1 E:T ratio: 40,000 cells NK cells versus 20,000 K562 cells per well. Cytotoxicity assay was performed for 4 h. The inhibition of statins on primary NK cell cytotoxicity could only be completely reversed by addition of 10 μM GGPP after rosuvastatin stimulation. Data was showed from 4 independent assays. Data analysis was performed by pair signed-rank test between statins only group and statins plus GGPP group.

Statins upregulated the LDLR expression (Figure 6A) without negative effects on cell viability (Figure 6B). As for NK-92 cells, higher LDLR expression levels enhanced VSV-G lentiviral transduction in the presence of GGPP (Figure 6C), without concerning the viability before or after transduction on primary NK cells (Figures 6B and 6D). Above all, among 5 kinds of statins, rosuvastatin was the most potent compound to both upregulate the LDLR on NK cells and increase the transduction. The LDLR and GFP were co-expressed in NK-92 (Figure 6E), while primary NK cells have more GFP cells when the LDLR was higher expressed (Figure 6F). After 36 h statins stimulation, the killing ability of primary NK cells in rosuvastatin was decreased. Fortunately, this suppressive effect was completely reversed by GGPP (Figure 7). Thus, a combination of rosuvastatin with GGPP in the culture medium augmented the transduction efficiency of primary NK cells.

## Discussion

Achieving high and effective transduction remains a big hurdle for the application of adoptive transfer of genetically modified NK cell therapies in the clinic.^7^ Numerous methods have been explored to improve lentiviral transduction efficiency.^33^ Here, we tested various statins and showed their enhancing effect on the transduction efficiency on NK cells by increasing the LDLR expression, receptor for VSV-G. The average increased ratio of the LDLR expression by statins both on NK-92 cells and primary human NK cells is approximately 1.5–3 times. The data in this study showed that, compared with the other statins, the lipophilic drug rosuvastatin most potently upregulated LDLR expression on NK cells, resulting in increased transduction efficiency. The addition of GGPP completely reversed the negative impact of statins on NK cell cytotoxicity.

The main biological function of statins is induction of LDLR expression on the cell surface of liver cells through inhibition of hepatic cholesterol synthesis.^34^ Upregulation of LDLR results in increased clearance of serum cholesterol. Clinical data shows a significant difference in prevention of coronary artery disease between patients treated with lipophilic or hydrophilic statins.^35^^,^^36^ The biological function of statins was shown to be dose-dependent with rosuvastatin seemingly having the highest activity.^37^ Here, we used statins to increase the LDLR expression to allow VSV-G pseudotyped viruses to enter the NK cell more efficiently. We observed the LDLR expression increasing is time course dependent of statins at 5 μM (Figure S1); however, LDLR is not dose dependent both in NK-92 (Figure 1B) and primary NK cells (Figure 4B). A similar study demonstrated that LDLR expression is not dose dependent.^38^ We also found no clear dose-dependent effects of the statins at the different doses tested (0.5 μM, 5 μM, and 20 μM). However, we found that high concentrations of statins (more than 20 μM) induced cell death, possibly due to negative effects on overall cell metabolism, thereby also inhibiting LDLR expression levels. Similarly, lower concentration of statins (<0.5 μM) may not be enough to induce LDLR high expression. The inhibitory effects on proliferation and cytotoxicity by statins have been reported in several studies.^28^^,^^29^ When compared with lipophilic statins (e.g., simvastatin and fluvastatin), hydrophilic statins (including pravastatin and rosuvastatin) showed milder or no suppression of proliferation and functional capacity on IL-2 stimulated NK cells (Figure 3).^39^ The viability of NK-92 cells in the medium control group before and after transduction decreased from 80% decrease to 70% (Figures 1A and 2C), while in primary NK cells, cell viability decreased from 75% to 65% (Figures 4A and 5A). Before transduction, were found no differences in cell viability in NK-92 cells or primary NK cells between the statin-treated group and the non-statin group. However, after VSV-G lentivirus transduction, the 20 μM of statins group showed negative effects on the viability of both NK-92 cells and primary NK cells. This effect is mainly due to the toxicity of VSV-G protein and protamine sulfate, with an additional detrimental effect of statins on NK cell viability. The inhibitory effects of statins on NK cell cytotoxicity could, among others, be due to the reduction of NK-target (tumor) cell adhesion, granule exocytosis (perforin and granzymes release).^26^^,^^27^ The release of cytotoxic lytic granules from NK cells is strongly dependent on phosphatidylinositol-specific phospholipase Cγ (PLCγ) phosphorylation and intracellular free calcium.^40^ Whether statins alter the abundance of intracellular Ca^2+^ in NK cells is debated: Raemer et al.^28^ showed that simvastatin had no influence on Ca^2+^ flux in human primary NK cells, while Poggi et al.^27^ demonstrated fluvastatin reduced intracellular free Ca^2+^ concentration in human NK cell clones. This discrepancy might be due to the different statins used or NK cell source variation. Furthermore, statins have been reported to change the killing components of NK cells, such as CD95L (FasL) and IFN-γ.^27^ In this study, we also investigated the CD107a degranulation, granzyme B, FasL expression, and IFN-γ secretion after co-culture of NK cells with statins (Figure S4). As previous studies^26^^,^^27^ already demonstrated, CD107a degranulation and FasL levels were reduced upon statin treatment. Interestingly, IFN-γ secretion was not altered. This could be due to cytokines and cell-cell contact with neighboring cells, which has been reported in earlier studies.^30^^,^^41^ These results could imply that the statin-induced inhibition of NK killing capacity might be due to changes in the degranulation process.

As described in the results section, statins decreased NK cell cytotoxicity. However, this effect could be reversed completely by GGPP.^28^ GGPP is synthesized by HMG-CoA reductase and is independent from the cholesterol metabolism. GGPP or mevalonate reversed the inhibitory effects of statins on HMG-CoA reductase.^29^ Crosbie et al. showed that GGPP or mevalonate was able to alter the cell cycle and DNA synthesis of NK cells, thus abrogating the negative effects of statins on NK cell proliferation.^29^^,^^42^

In addition to statins, other approaches have also been tested to improve high NK cell transduction to create CAR-NK cells.^43^ Polycations like polybrene, dextran, poly-L-lysine, and protamine sulfate are supposed to eliminate the charges on cell membranes, thereby enhancing viral transduction efficiency.^15^^,^^44^ In a direct comparison of polybrene, protamine sulfate, and dextran sulfate,^15^ it was shown that dextran-treated NK cells show the highest transduction efficiency at 38% GFP-positive cells, whereas no GFP expression was detected in cells treated with either polybrene or protamine sulfate. In contrast to the present study, their research showed that 8 μg/mL dextran had no influence on the viability and killing capacity of NK cells, while we observed that this concentration of dextran was detrimental for NK cell viability (below 20%; Figure 1A). This difference might be due to the use of freshly isolated NK cells, while Nanbakhsh et al. used expanded NK cells.^15^ The second approach to increase higher NK cell transduction efficiency is by cytokine or mitogen stimulation. Previous studies demonstrated that LDLR upregulation, in conjunction with enhanced proliferation and cytotoxicity, can also be achieved by stimulation with IL-2.^45^ Soluble IL-2 plus IL-12 stimulation enhances VSV-G lentiviral transduction, which could be further enhanced by 1 μg/mL PHA.^21^ Culturing primary human NK cells on K562 feeder cells with expressing membrane-bound IL-21 and 4-1BBL also augmented gene transduction by 50%.^46^^,^^47^ Third, suppression of intracellular antiviral defense mechanisms are also described to increase lentiviral transduction of NK cells. BX795, which is an inhibitor of the TBK1/IKKe complex that controls antiviral responses, is able to boost lentiviral gene transduction efficiency by 3.8-fold.^12^ Next, higher transduction efficiency may be achieved with other pseudotypes of viruses, like alpharetroviral vectors^48^ or baboon envelope pseudotyped lentivirus.^47^ Finally, methods have also been developed to obtain genetically engineered NK cells from HSC^49^ or induced pluripotent stem cells.^50^

Statins also have anti-inflammatory and immunomodulatory properties in clinical treatment.^51^ Given that statins facilitate the entry of lentiviruses into cells, this could render patients more susceptible to virus infection. Even though some small studies indicate that statins indeed promoted virus infection or activity (e.g., in herpes zoster^52^ and respiratory viral infections^53^), other studies indicate that statins restrict virus activity (e.g., in HIV^54^ and Ebola infection^55^), while a third group shows no effect.^53^^,^^56^ Most retrospective studies have intrinsic methodological limitations and too few relevant randomized controlled trials have been performed on relation between statins and viral infectious incidence.^56^ Altogether, at this time there is no body of evidence that allows us to draw definitive conclusions on the effects of clinical statin use on virus infections.

In the current paper, we describe a novel strategy to improve the transduction on human NK cells by increasing the expression of the LDLR. NK cells expressing higher LDLR levels on the surface could get easier transduced with VSV-G lentivirus by helping the VSV lentivirus entry into the NK cell (Figures 2F and 5D). Now the structural composition of how VSV-G recognizes the LDLR on the cell surface has been elucidated,^57^ we can imagine that specific overexpression of the cysteine-rich domains (CR2 or CR3) of the LDLR on NK cells could enhance the transduction efficiency. The limitation of the approach is that human primary NK cells express low levels of LDLR. However, other VSV-G receptors may be present on NK cell surface, like the leucine-rich repeat-containing G protein-coupled receptor 4 (Lgr4),^58^ HSP90B1,^59^ and LDL-receptor other family members.^17^

For future therapeutic applications, rosuvastatin plus GGPP currently is the most potent combination that increases VSV-G lentivirus transduction efficiency without a reduction of NK cell cytotoxicity. This finding is important for the both scientists and clinicians, as it facilitates the transduction of NK cells that are known to be hard to transduce, but it holds important promise for cancer adoptive cell therapy.

## Materials and Methods

### Cell Lines and Culture

NK-92 cells (CRL-2407, ATCC, Manassas, VA, USA) were cultured in alpha Minimum Essential medium (Thermo Fisher Scientific, Waltham, MA, USA) without ribonucleosides and deoxyribonucleosides, supplemented with 2 mM L-glutamine, 2.2 g sodium bicarbonate (Sigma-Aldrich, Munich, Germany), 0.2 mM inositol (Sigma-Aldrich), 0.1 mM 2-mercaptoethanol (Sigma-Aldrich), 0.02 mM folic acid (Sigma-Aldrich), 100 U/mL recombinant IL-2 (Proleukin, Novartis, Basel, Switzerland), 12.5% horse serum (ATCC), 12.5% fetal calf serum (FCS, Greiner Bio-One, Frickenhausen, Germany), and 1% penicillin/ streptomycin (Thermo Fisher Scientific). K562 (ATCC CCL-243) cells were cultured in IMDM supplemented with 10% FCS (Greiner Bio-One) and 1% penicillin/streptomycin (Thermo Fisher Scientific). 293FT cells (R700-07, Thermo Fisher Scientific) were cultured in DMEM/high glucose medium supplement with 10% FCS (Greiner Bio-One), 0.1 mM MEM non-essential amino acids (NEAA, Thermo Fisher Scientific), 2 mM L-glutamine (Thermo Fisher Scientific), and 1 mM MEM sodium pyruvate (Thermo Fisher Scientific). The Jurkat cell line (ACC 282, DSMZ, Braunschweig, Germany) was cultured in RPMI-1640 medium (Thermo Fisher Scientific) with 10% FCS and 1% penicillin/streptomycin.

### Human NK Cell Isolation and Activation

Primary human NK cells were isolated from anonymous buffy coats (Sanquin, Maastricht, the Netherlands). The use of buffy coats, being a byproduct of a required Medical Ethical Review Committee (METC) procedure, does not need ethical approval in the Netherlands under the Dutch Code for Proper Secondary Use of Human Tissue. NK cells were subsequently isolated by negative selection with an NK cell isolation kit (130-092-657, Miltenyi Bbiotec, Bergisch Gladbach, Germany) using MACS beads as previously described.^60^ For short-term activation, NK cells were cultured in RPMI-1640 medium (GIBCO) supplemented with 10% fetal calf serum (Greiner Bio-One) and 100 U/mL penicillin-streptomycin (GIBCO). NK cells were activated overnight with 1,000 IU/mL recombinant human IL-2. All cells were cultured with 5% CO_2_ at 37°C in a humidified cell culture Sanyo MCO-20AIC incubator (Sanyo Electric, Osaka, Japan).

### Co-culture of NK Cells with Statins and Non-Statin Compounds

NK-92 cells were seeded at 0.1 × 10^6^ cells/mL in round-bottom 96-well plates (3799, Corning Life Sciences B.V., Amsterdam, the Netherlands). Atorvastatin (10493, Cayman Chemical), fluvastatin (10010337, Cayman Chemical), pravastatin (10010342, Cayman Chemical), rosuvastatin (12029, Cayman Chemical), and simvastatin (10010344, Cayman Chemical) were dissolved in DMSO (Sigma-Aldrich). Statins were used at final concentration of 20 μM, 5 μM, and 0.5 μM. DMSO was diluted in the same volume and served as solvent control. IL-2, IL-21 (Thermo Fisher Scientific), vitamin C (Sigma-Aldrich), dextran (Sigma-Aldrich), prostaglandin E2 (PGE2, Sigma-Aldrich), protamine sulfate (Sigma-Aldrich), and vectofusin-1 (Miltenyi Biotec) were dissolved in distilled water (GIBCO). DMSO was added 0.8 μL, 0.2 μL, and 0.02 μL, respectively, at the same volume as the statin group in 96-well plates. IL-21 was added at 20 ng/mL, 5 ng/mL, and 0.5 ng/mL. Vitamin C was used at concentrations of 500 μg/mL, 50 μg/mL, and 5 μg/mL as described previously.^23^ Dextran was used at 80 μg/mL, 8 μg/mL, and 0.8 μg/mL.^15^ PGE2 was used at 100 μM, 10 μM, and 1 μM.^22^ Vectofusin-1 was used at 50 μg/mL, 5 μg/mL, and 0.5 μg/mL.^14^^,^^61^ GGPP ammonium salt was purchased from Sigma-Aldrich and was added at 10 μM in co-culture assays.^29^

### Vectors and Lentivirus Production and NK Cells Transduction

All the virus procedures were under the surveillance by the Center for Research Innovation, Support and Policy (CRISP) of Maastricht University Medical Center (Genetic Modification License number: GGO-00-177). pCDH-EF1-copGFP-T2A-Puro was a gift from Kazuhiro Oka (Addgene plasmid # 72263, Watertown, MA, USA). pRSV-Rev (Addgene # 12253), pMDLg/ pRRE (Addgene # 12251), and pMD2.G (Addgene # 12259) were gifts from Didier Trono.^62^ Plasmids were expanded in Stbl3 *E. coli* (Thermo Fisher Scientific) and prepared by followed the instruction of NucleoBond Xtra Midi Kit (Macherey-Nagel GmbH, Düren, Germany). All these four plasmids were mixed with the ratio 5:2:2:1 in 40 μg and were transfected in one 150 mm cell culture dish of 293FT cells in the presence of 80 μg polyethylenimine (PEI, Polysciences, Warrington, PA, USA). After 48 h, every 24 h and continually for 5 days, virus supernatant was collected and filtered through a 0.45 μm syringe filter (Merck Millipore, Burlington, MA, USA). Pooled virus supernatant was concentrated using Lenti-X Concentrator (Takara, Saint-Germain-en-Laye, France) according to the manufacturer’s protocol. Viral titers were determined using Jurkat cells by performing 2 × serial dilutions and detection of GFP expression using flow cytometry 48 h post-transduction. VSV-G lentiviruses were added to cells at a MOI 10 in the presence of 10 μg/mL protamine sulfate after 36 h of statin co-incubation.

### Flow Cytometry and Antibodies Staining

For flow cytometric analysis, cells were stained with Live/Dead Fixable Aqua Dead Cell Stain Kit (Thermo Fisher Scientific) in PBS on ice for 30 min. Then cells were stained using the following antibodies recognizing human antigens: CD3 (Clone OKT3, BD Biosciences, San Jose, CA, USA), CD56 (Clone REA196, Miltenyi Biotec), LDLR (Clone C7, BD Biosciences), CD95L (Clone NOK-1, BD Biosciences), CD107a (Clone H4A3, Miltenyi Biotec), granzyme B (Clone REA226, Miltenyi Biotec), and IFN-γ (REA600, Miltenyi Biotec). Antibodies mixture in fluorescence-activated cell sorting (FACS) staining buffer (PBS + 1% FCS) were added to NK cells and incubated at 4°C for 30 min. Fluorescence was read on a BD FACS CantoII flow cytometer. Data were analyzed with FlowJo 10.1 (TreeStar, Ashland, OR, USA) software.

### Cytotoxicity Assay

The NK cell killing ability against tumor cells was determined in a 4 h flow cytometry-based assay. Tumor cells were pre-labeled with Cell Tracker Deep Red Dye according to the manufacturer’s protocol (Thermo Fisher Scientific) and were cultured overnight. Tumor cells were harvested and seeded at 20,000 cells per well in round-bottom 96-well plates. NK cells were seeded in duplicates with different effector to target ratios (E/T ratio) and cultured in RPMI-1640 medium for 4 h. After 4 h, dead Deep Red-labeled tumor cells were measured with Live/Dead Fixable Aqua Dead Cell Stain Kit (Thermo Fisher Scientific) by flow cytometry. Specific cytotoxicity was calculated as previously described.^60^

### Statistical Analysis

All statistical tests used in this study were completed with GraphPad Prism 8 software (Graphpad Software, San Diego, CA, USA). The specific statistical tests used for each comparison are specifically annotated in the figure legends, respectively. For performing multiple comparisons, we used two-way ANOVA and Bonferroni post-tests comparing to the medium control group. For cell viability, Mann-Whitney U test was used to compare with the medium control group. ∗p < 0.05, ∗∗p < 0.01, and ∗∗∗p < 0.001. Results were considered non-significantly (n.s.) different if p ≥0.05.

## Author Contributions

Y.G., R.G.J.K.W., and I.J. designed and performed experiments. R.G.J.K.W. and W.T.V.G. evaluated data. A.J.G. contributed protocols and reagents. Y.G., R.G.J.K.W., and W.T.V.G. wrote the manuscript. G.M.J.B. and W.T.V.G. conceived and designed the study, supervised the work, and wrote the manuscript. All authors read and approved the final manuscript.

## Conflicts of Interest

G.M.J.B. and W.T.V.G. are the founders of CiMaas BV. All other authors declare no competing interests.

## Supplemental Information

Document S1. Figures S1–S6 and Supplemental Methods

Document S2. Article plus Supplemental Information
