# Supplementary material for: Rosuvastatin Enhances VSV-G Lentiviral Transduction of NK Cells via Upregulation of the Low-Density Lipoprotein Receptor
Source: Mol Ther Methods Clin Dev. 2020 Mar 29;17:634–46. doi: 10.1016/j.omtm.2020.03.017 (PMC7150439; doi:10.1016/j.omtm.2020.03.017)
Supplement: Document S1. Figures S1–S6 and Supplemental Methods [file mmc1.pdf]

OMTM, Volume *17*

Supplemental Information

Rosuvastatin Enhances VSV-G Lentiviral Transduction of NK Cells via Upregulation of the Low-Density Lipoprotein Receptor

Ying Gong, Roel G.J. Klein Wolterink, Ian Janssen, Arjan J. Groot, Gerard M.J. Bos, and Wilfred T.V. Germeraad

Supplemental Figures

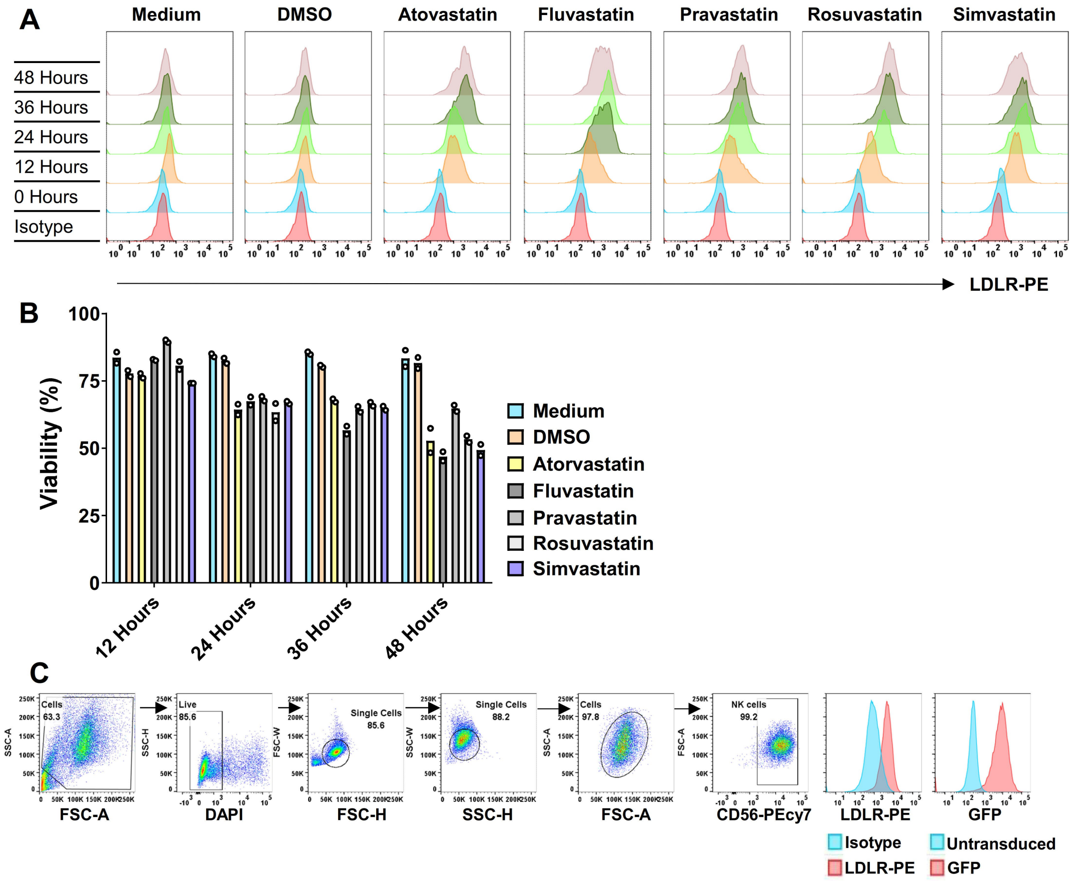

Figure S1 Time course of LDLR expression after statin stimulation.
0.1 x 10^6 cell/mL NK-92 cells were cultured with 5 µM statins for different periods. (A) LDLR expression levels are upregulated by statins in a time-dependent manner Histograms show one representative sample of LDLR expression levels analyzed using flow cytometry. (B) Prolonged culture with statins has a negative impact on NK-92 cell viability. Dead cells were stained with Fixable live/dead Aqua V500. Data shown is one representative of 3 independent assays. (C) Gating strategy of LDLR and GFP expression on NK-92 cells.

A

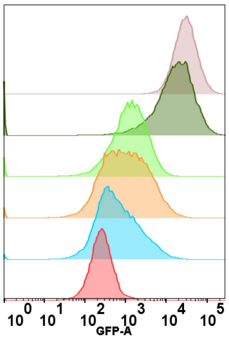

NK92 cells transduced with Protamine Sulfate			
**MOI**	**GFP MFI**	**GFP (%)**	**Viability (%)**
**25**	35334	99,8	75,5
**20**	23655	95,8	72,8
**15**	1837	76,6	74,7
**10**	2123	62	79,5
**5**	1390	47,2	83,4
**Untransduced**	271	4,3	91,6

B

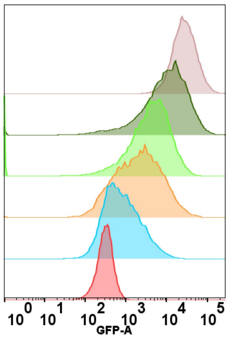

NK92 transduced with Protamine Sulfate + 5μM Rosuvastatin			
**MOI**	**GFP MFI**	**GFP (%)**	**Viability (%)**
**25**	33339	99,7	73,9
**20**	16002	94,6	75,1
**15**	6063	88,9	75,9
**10**	4247	79,8	81,3
**5**	1396	56,9	86,8
**Untransduced**	329	4,93	94,1

C

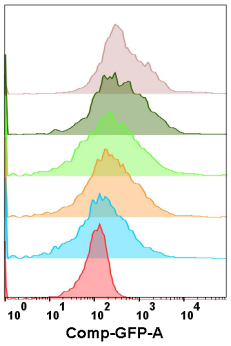

Primary NK transduced with Protamine Sulfate			
**MOI**	**GFP MFI**	**GFP (%)**	**Viability (%)**
**25**	1222	45,60	51,00
**20**	1159	40,70	47,75
**15**	868	27,10	65,50
**10**	627	27,65	74,45
**5**	437	18,20	79,00
**Untransduced**	118	1,12	80,30

D

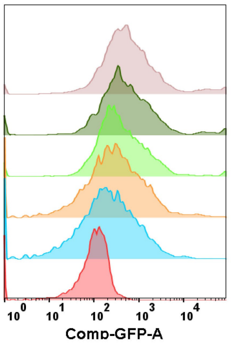

Primary NK transduced with Protamine Sulfate + 5μM Rosuvastatin			
**MOI**	**GFP MFI**	**GFP (%)**	**Viability (%)**
**25**	2580	56,40	46,70
**20**	2024	50,05	50,70
**15**	2120	42,90	62,35
**10**	1014	33,75	77,70
**5**	680	26,75	82,60
**Untransduced**	119	1,35	80,90

Figure S2 Higher virus concentrations promote GFP expression at the expense of cell viability. NK-92 were incubated without (A) or with (B) 5 μM rosuvastatin for 36 hours at 0.1 x 10^6 cells/mL. Different MOI of VSV-G lentivirus were added in the NK-92 cells in the presence of 10 μg/mL protamine sulfate. Histograms and tables show GFP expression (MFI and proportion of positive cells) and cell viability 48 hours after transduction. Similarly GFP expression levels and viability are shown for primary NK cells following lentiviral transduction without (C) or with (D) rosuvastatin pre-incubation.

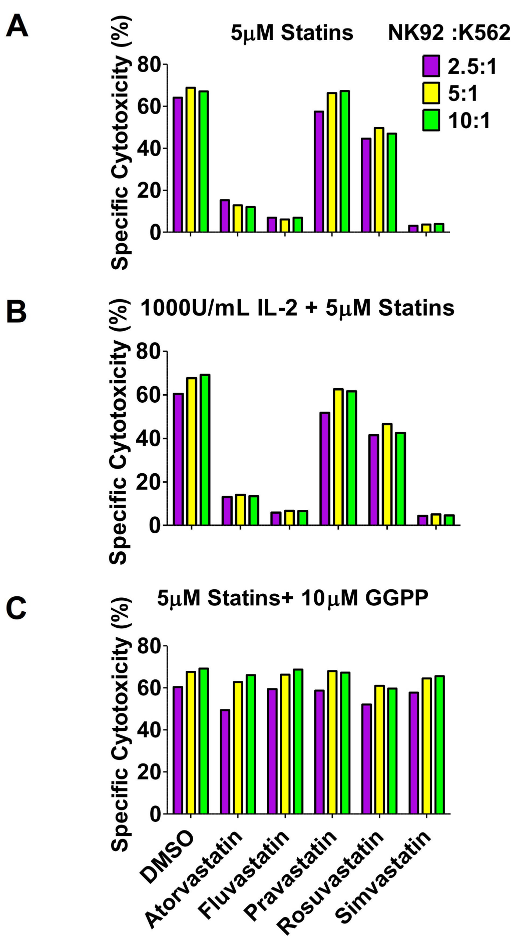

Figure S3 GGPP but not IL-2 can completely reverse the negative impact of statins on the cytotoxic capacity of NK-92 cells.

(A) NK-92 cell cytotoxicity was suppressed by lipophilic statins after 36 hours incubation. However, NK-92 cell cytotoxicity was not changed after pravastatin and not completely be restrained by rosuvastatin. NK-92 cells were seeded at a density of 0.1 X 10^6 cells/mL in 12 well plates for statin stimulation. After removing the supernatant, NK-92 cells were added according to indicated E:T ratio in 96 well-plates. 20,000 K562 cells were seeded per well as target cells. Cytotoxicity assay was performed for 4 hours with different E:T ratios. (B) Statin (5 μM) induced inhibition of NK cell cytotoxicity could not be reversed by 1000 U/mL IL-2. (C) GGPP completely reversed the cytotoxicity suppression of statins on NK-92 cells. Data is shown of one representative assay of 3 independent assays as mean cytotoxicity.

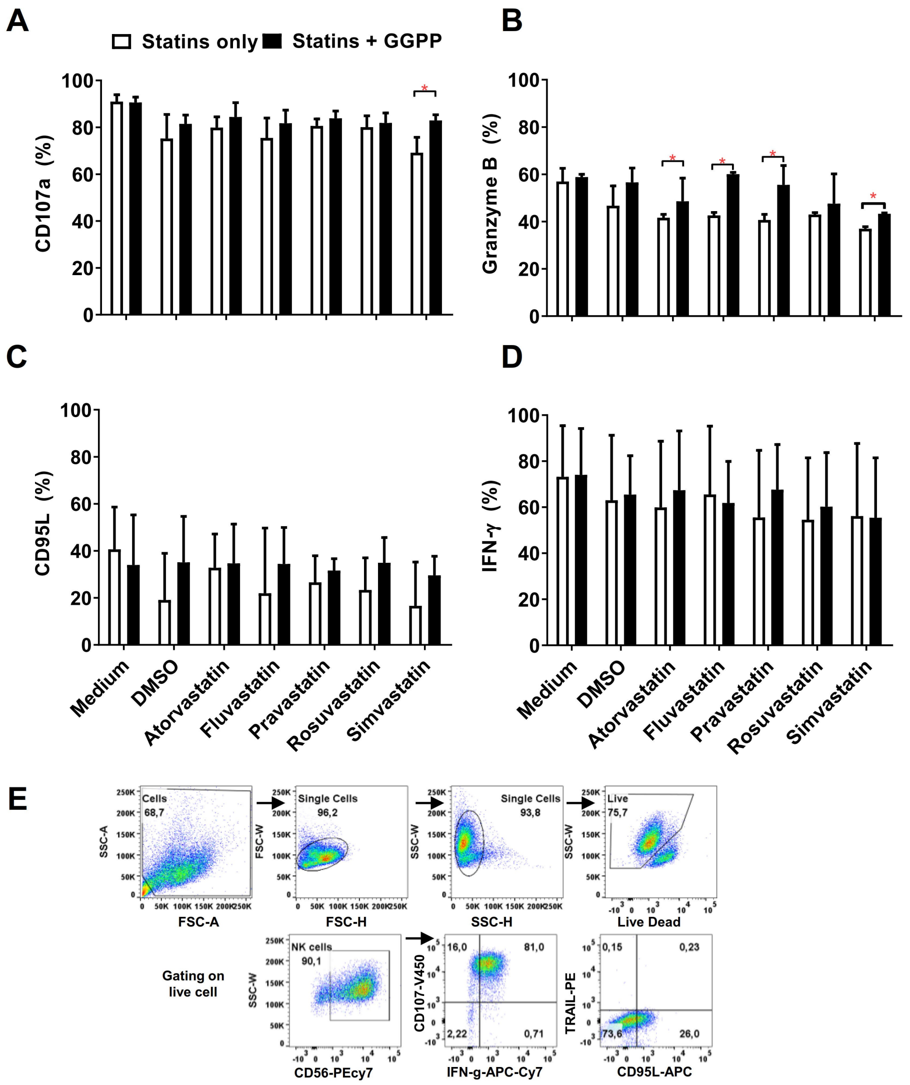

Figure S4 Statins have a negative effect on CD107a and granzyme B expression, which can be reversed using GGPP.

0.1 x 10^6 NK-92 cells were cultured in 1mL with 5 μM statins for 36 hours. Medium was replaced with medium containing CD107a - V450 antibody, and PMA (20 ng/mL) plus ionomycin (1 μg/mL). After 1 hour pre-incubation, 10 μg/mL brefaldin A was added. Cells were stained with surface markers (CD56 and CD95L), and subsequently fixed and permeabilized, followed by IFN-γ intracellular staining. CD107a (A), Granzyme B (B), CD95L (C) and IFN-γ (C) expression in NK-92 were analyzed by flow cytometry. Compared with medium and DMSO, only simvastatin down-regulated CD107a, but was reversed by GGPP. All the statins inhibit the granzyme B, except for rosuvastatin. Fortunately, this negative effect was reversed by adding 10 μM GGPP. (E) Gating strategy of CD107a, CD95L and IFN-γ on NK-92. Data is shown as mean ± SD from N=3 independent experiments performed at different times. Data analysis was performed using a pair signed-rank test between the statins only group and the statins plus GGPP group.

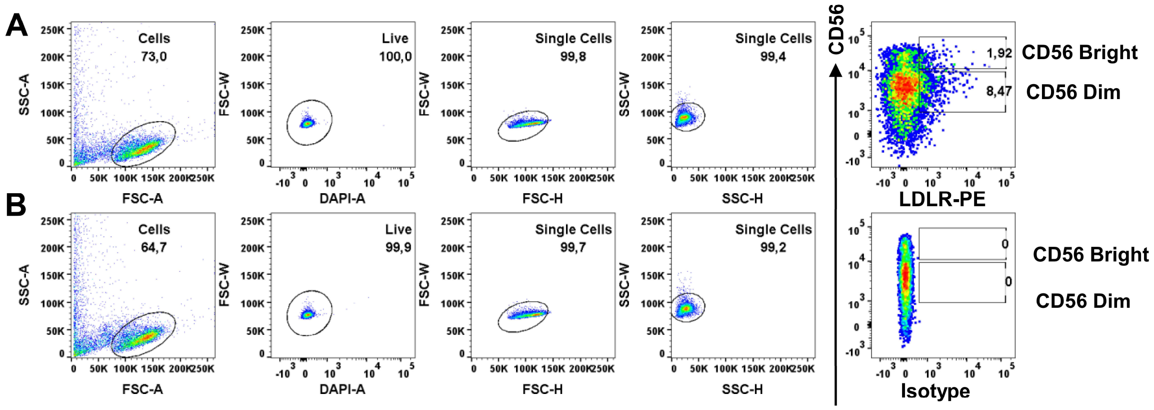

Figure S5 LDLR expression levels on fresh isolated human primary NK cell.
Human NK cells were isolated using negative magnetic selection. LDLR expression levels were analyzed for the CD56 bright and CD56 dim sub-populations using flow cytometry.

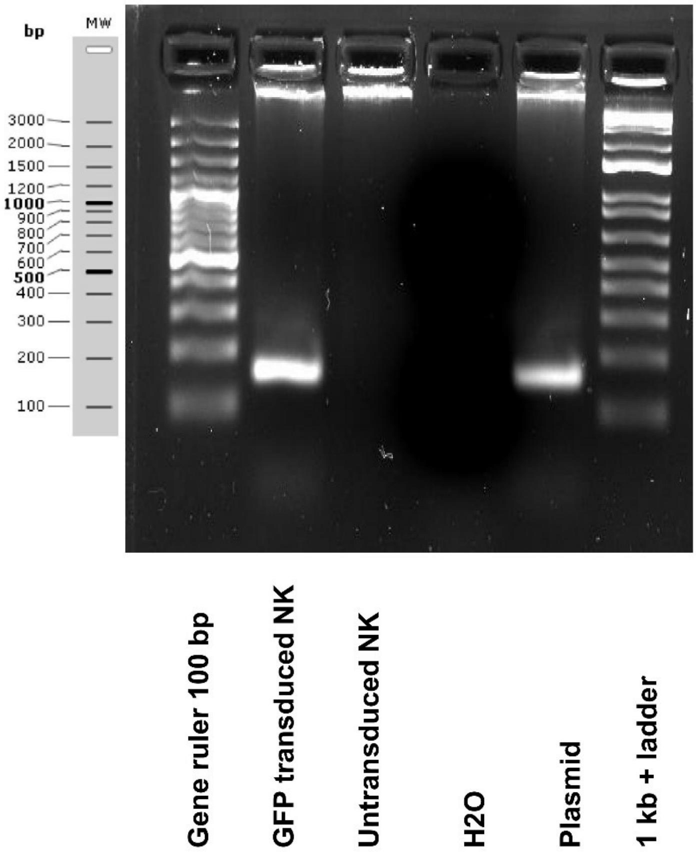

Figure S6 Lentiviral integration in human primary NK cells.
Genomic DNA was extracted 48 hours after VSV-G lentiviral transduction of human primary NK cells. One pair of primer specific amply the GFP fragment with a size at 157 bp. PCR product was running on 1.2 % agarose TAE gel (70 V, 45 minutes). A plasmid encoding GFP was used as positive control, H2O is used as the ‘no template’ control. Untransduced NK cells were used as a negative control.

Supplemental Methods

CD107a degranulation assay

To evaluate NK cell activation by tumor cells or after stimulated with Phorbol 12-myristate 13-acetate (PMA, Sigma Aldrich) plus ionomycin (Sigma Aldrich), CD107a expression on NK cells was analyzed through flow cytometry. NK cells were incubated with PMA (20 ng/mL) plus ionomycin (1 μg/mL) with 2 μL anti-CD107a-Horizon V450 (H4A3, BD Biosciences) in a 96-well plate. After 1 hour of co-culture, 10 μg/mL Brefeldin A (BFA, BD Biosciences) was added. After another 3 hours, the plate was placed on ice to stop the reaction. NK cells were further stained with anti-human CD3 and CD56 antibodies conjugated with appropriate fluorophores surface markers as described above.

Intracellular staining

For intracellular staining of interferon-gamma (IFN-γ), cells were stimulated with PMA (20 ng/mL) plus ionomycin (1 μg/mL) and 10 μg/mL BFA in the culture medium for 4 hours. Then cells were harvested, washed, and stained with fluorochrome-conjugated anti human CD3 and CD56 surface monoclonal antibodies for 30 minutes at 4 °C. Cells were then washed twice and treated with fixation buffer (eBiosciense, Thermo Scientific) for 15 minutes at room temperature. After washing, cells were permeabilized in permeabilization buffer for 30 minutes at 4 °C degree. After washing, fixation and permeabilization, cells were stained with fluorochrome-conjugated anti human IFN-γ antibody (Clone: 45-15, Miltenyi) anti human Granzyme B (Clone REA226, Miltenyi Biotec). After 2 more washes, the cells were analyzed by FACS Canto II (BD Biosciences).

Genomic extraction and PCR

48 hours after transduction, 0.5 x 10^6 primary NK cells were harvested. Primary genomic DNA was isolated using Lucigen QuickExtract™ DNA Extraction Solution (QE0905T, Lucigen Corporation, Wisconsin, USA) as published elsewhere [1]. The GFP construct was amplified using PrimeSTAR HS polymerase kit (R010A, Takara Bio) and the following primers: GFP-forward gtgatgggctacggcttctacc and GFP-reverse primer cctcgtagcggtagctgaag.

Supplemental References

1. Bak, R.O., and Porteus, M.H. (2017). CRISPR-Mediated Integration of Large Gene Cassettes Using AAV Donor Vectors. Cell reports *20*, 750-756.
