# Supplementary material for: Rosuvastatin Enhances VSV-G Lentiviral Transduction of NK Cells via Upregulation of the Low-Density Lipoprotein Receptor
Source: Mol Ther Methods Clin Dev. 2020 Mar 29;17:634–46. doi: 10.1016/j.omtm.2020.03.017 (PMC7150439; doi:10.1016/j.omtm.2020.03.017)
Supplement: Document S2. Article plus Supplemental Information [file mmc2.pdf]

Molecular Therapy
Methods & Clinical Development
Original Article

Rosuvastatin Enhances VSV-G Lentiviral Transduction of NK Cells via Upregulation of the Low-Density Lipoprotein Receptor

Ying Gong,[1,2] Roel G.J. Klein Wolterink,[1,2] Ian Janssen,[1,2] Arjan J. Groot,[2,3] Gerard M.J. Bos,[1,2,4] and Wilfred T.V. Germeraad[1,2,4]

[1]Department of Internal Medicine, Division of Hematology, Maastricht University Medical Center+, Maastricht, the Netherlands; [2]GROW - School for Oncology and Developmental Biology, Maastricht University, Maastricht, the Netherlands; [3]Department of Radiology, Maastricht University, Maastricht, the Netherlands; [4]CiMaas BV, Maastricht, the Netherlands

Adoptive natural killer (NK) cell therapy is attaining promising clinical outcomes in recent years, but improvements are needed. Genetic modification of NK cells with a tumor antigen-specific receptor on their surface coupled to intracellular signaling domains may lead to enhanced cytotoxicity against malignant cells. One of the most common approaches is by lentivirus-mediated transduction. However, NK cells are difficult to transduce and various methods have been attempted with different success rates. Because the low-density lipoprotein-receptor (LDLR) is the receptor of vesicular stomatitis virus (VSV) and is expressed only at low levels on NK cells, we tested the potential of 5 statins and 5 non-statin compounds to increase the LDLR expression, thereby facilitating viral transduction. We found that the transduction efficiency of VSV-G pseudotyped lentivirus is augmented by statins that induced higher LDLR expression. In both NK-92 cells and primary NK cells, the transduction efficiency increased after treatment with statins. Furthermore, statins have been reported to suppress NK cell cytotoxicity; however, we showed that this can be completely reversed by adding geranylgeranyl-pyrophosphate (GGPP). Among the statins tested, we found that the combination of rosuvastatin with GGPP most potently improved viral transduction without affecting the cytotoxic properties of the NK cells.

INTRODUCTION

Cancer immunotherapy, with its higher specificity and fewer side effects compared to traditional anti-cancer therapies, has become an important tumor therapeutic strategy.[1] The main purpose is to break tolerance and revitalize the body's immune system that has become insensitive for advanced malignancies. The most prominent advantage of immunotherapy is the potential ability to eradicate distant metastases leading to a possible cure in a percentage of patients. A sophisticated form of immunotherapy is cellular therapy that includes dendritic cell vaccination, adoptive therapy of tumor infiltrate lymphocytes (TIL), tumor-specific T cell receptor T cells (TCR-T), and natural killer (NK) cells, as well as chimeric antigen receptor (CAR)-T and CAR-NK.

NK cells are innate immune cells having a surveillance function to eradicate virally infected cells or malignant cells. NK cells have several cytotoxic components leading to destruction of target cells. Perforin and granzymes incorporate into granules in the cytoplasm once NK cells are educated. After NK cells have recognized and bound target cells, the granules merge with the cell membrane, and they secrete their content to mediate the killing process of the target cell by inducing apoptosis along the caspase pathways. One of the main mechanisms of NK cells to become active and cytotoxic is the concept of "missing self." NK cells can recognize cells that are missing major histocompatibility complex (MHC) class I and thereby become activated. Moreover, NK cells can directly recognize tumor cells independent of MHC presentation like T cells.[2] The other mechanisms by which NK cells kill, include the death receptors CD178 (Fas Ligand [FasL]) and tumor necrosis factor-related apoptosis-inducing ligand (TRAIL), also leading to apoptosis, but are different in terms of timing.[3]

Adoptive transfer of mature alloreactive NK cells was shown to be effective in the treatment of patients with acute myeloid leukemia (AML) and to prevent relapse.[4] In contrast to the percentage of T cells, NK cells comprise a relatively small population and are not persistent as long as T cells *in vivo*.

With the development of genetic modification methods and promising clinical outcome of gene engineered T cells, NK cells could be great effector cells once armed with a specific antigen ligand or antibody. Building on the first clinical successes and subsequent

Received 28 February 2020; accepted 23 March 2020;
https://doi.org/10.1016/j.omtm.2020.03.017.

Correspondence: Wilfred T.V. Germeraad, PhD, Department of Internal Medicine, Division of Hematology, Maastricht University Medical Center+, Maastricht, the Netherlands.
E-mail: w.germeraad@maastrichtuniversity.nl

 © 2020 The Author(s).
This is an open access article under the CC BY license (http://creativecommons.org/licenses/by/4.0/).

application of the CD19 CAR-T for B cell hematological malignancies, there are increasing CAR-T or, more recently, CAR-NK clinical trials in progress to treat cancer patients.[5,6]

Generating CAR-T or CAR-NK cells consists of a genetic modification of cells resulting in surface expression of the antigen binding part of an antibody coupled to intracellular T cell or NK cell signaling molecules. This structure endows the T cell or NK cell to directly recognize the native tumor antigen,[7] resulting in stronger cell activation and enhanced cytotoxicity.

Despite the sensational clinical results with CAR-T cells, including their long *in vivo* persistence, it comes with the potential of various side effects; especially a cytokine release storm and neurotoxicity may cause dramatic outcomes and even death.[8] In this concept, NK cells with their short lifespan and high killing capacity could form an alternative and effective cell therapy.[4] Furthermore, combining a best-of-both-worlds concept, a CAR-NK cell can be generated.

Genetic modification to generate CAR-NK cells is aimed to improve their killing ability and tumor antigen targeting capacity. However, high efficiency of transfection or transduction of NK cells remains a big challenge. Retroviruses or lentiviruses are the transfer methods of choice to obtain permanent integration of the transgene with high transduction efficiencies. Numerous reagents have been used to enhance viral transduction. Protamine sulfate or polymers (dextran or polybrene) can eliminate the electronic charge on the cell membranes.[9] Cyclosporine A[10] and rapamycin relieve distinct lentiviral restriction blocks in hematopoietic stem and progenitor cells.[11] Tolga et al.[12] reported that inhibition of intracellular antiviral defense mechanisms augments lentiviral transduction of human NK cells. Vectofusin-1[13] and prostaglandin E2[9] and dextran[11] have been reported to enhance lentiviral vector transduction of human hematopoietic stem cells (HPSCs), T lymphocytes,[14] and primary NK cells,[15] respectively, without further mechanistic description.

Vesicular stomatitis virus G protein (VSV-G) can be used as an envelope protein on the lentiviral particles,[16] and the low density lipids (LDL) receptor and its family members serve as the cellular VSV receptors in human primary lymphocytes.[17] Upregulation of the LDL receptor on lymphocytes may improve the VSV-G lentiviral transduction.[18] Interestingly, various groups have shown that the expression levels of LDLR in human B and T lymphocytes can be increased using antibodies, cytokines, and estrogen receptor modulators.[18,19] Clinicians used statins as anti-hyperlipidemia drugs because they will upregulate the LDL receptor on endothelial cells thereby increasing lipid removal from the blood. However, in NK cells, the impact of LDLR expression and its modulators has not been investigated. Therefore, we investigated which compounds influence the LDLR expression levels on NK cells and how LDLR expression levels improve lentiviral transduction efficiency of NK cells while NK cells ultimately maintain their cytotoxic capacity.

RESULTS

Statins Enhance LDLR Expression Levels in the NK-92 Cell Line

Given that LDLR expression levels in human B and T lymphocytes can be influenced using compounds compatible with *in vitro* culture, we first asked what drugs influence LDLR expression levels in human NK cells. For screening purposes, we made use of the human NK cell line NK-92. This cell line shares important features with primary NK cells: it recognizes viruses and tumor cells, has cytotoxic capabilities, and produces characteristic NK cell cytokines.[20] Based on previous publications, we tested compounds that have been reported to enhance NK cell transduction (interleukin-21 [IL-21][21] and dextran[15]), enhance lentiviral transduction in hematopoietic stem cells and T lymphocytes (vectofusin-1[14] and prostaglandin E2[22]), and promote NK cell proliferation (ascorbic acid).[23] Furthermore, we tested statins (high-mobility group-coenzyme A [HMG-CoA] reductase inhibitors) that are clinically used as lipid-lowering medication[24] and that have been reported to directly increase *LDLR* mRNA in human mononuclear cells.[25] Here, we examined the effects of three lipophilic statins (atorvastatin, fluvastatin, and simvastatin) and two hydrophilic statins (pravastatin and rosuvastatin).

Previously, Hillyard et al.[26] had demonstrated that 10 μM statin is detrimental for the NK cell membrane raft, which is a key functional component for NK cell cytotoxicity. The same statins concentration was also shown by Poggi et al.,[27] who reported that 10 μM fluvastatin was able to decrease the activation markers on NK cells. Initially, we tested three different concentrations of statins: 0.5 μM, 5 μM, and 20 μM. Higher doses of 50 μM resulted in a 50% reduction in viability after 36 h incubation and were therefore not used (data not shown). Culture of NK-92 cells with the various statins at these concentrations did not have a significant negative impact on cell viability (Figure 1A). Similarly, most non-statin compounds, except dextran, did not negatively impact NK-92 cell viability, even at the highest concentrations tested (Figure 1A).

Flow cytometric analysis showed that all statins tested increased the LDLR protein expression at least 3-fold, while none of the other compounds significantly upregulated LDLR expression levels (Figures 1B and 1C). LDLR expression levels were not strictly dose-dependent: we observed that LDLR expression levels were generally highest following culture in 5 μM of the respective statins (Figure 1C), without a negative impact on cell viability (Figure 1A). Therefore, we determined 5 μM to be the optimal concentration to augment the expression of LDLR on NK-92 cells. Statins at higher concentrations inhibit the viability of NK cells, whereas at a low 0.5 μM LDLR expression was not highly induced. The major impact of the statins is apparent within 12 h (Figure S1A) and the induction of LDLR expression on NK-92 cells following statin stimulation is time-dependent. Moreover, addition of statins also impacted on cell viability, as 48 h after statin administration, the NK cells that normally grow in clumps loosened, resulting in a gradual decrease in overall viability (Figure S1B), while LDLR expression levels did not increase further (Figure S1A). Therefore, we determined 5 μM to be the optimal

concentration and 36 h incubation for statins to upregulate LDLR expression in NK-92 cells, since LDLR expression was highest at this concentration with no negative impact on cell viability (Figures S1B and S1C).

Statins Enhance the Viral Transduction Efficiency of NK-92 Cells

Next, we determined whether the statin-induced increase of cell surface LDLR expression levels in NK-92 cells led to enhanced VSV-G lentiviral transduction efficiency. Thus, we treated cultured NK-92 cells with statins for 36 h, followed by lentiviral transduction with a GFP-encoding vector and analysis after 48 h (Figures 2A and 2B). We used flow cytometric analysis of the fraction of GFP-positive cells and their mean fluorescence index (MFI) to determine the lentiviral transduction efficiency. As reported by other groups,[7,21] lentiviral transduction of NK-92 cells has a mild negative impact on cell viability (Figure 2C). However, in combination with lentiviral transduction, most statins unexpectedly had a profound impact on cell viability. Notably, this effect was less pronounced in the cultures treated with the hydrophilic statins pravastatin and rosuvastatin compared with the lipophilic statins (Figure 2C). Importantly, atorvastatin, rosuvastatin, and simvastatin significantly increased the fraction of GFP-expressing cells and the GFP expression levels 1.5- to 2.5-fold, indicating improved transduction efficiency (Figures 2D and 2E). In the non-statins group, only dextran promoted transduction efficiency at the high expense of cell viability. Interestingly, higher doses of statins did not further enhance GFP expression levels after lentiviral transduction (Figure 2E), while the observed negative effects of statins on cell viability of lentivirus transduced NK-92 cells were dose-dependent (Figure 2C). Pooled analysis of the effect of LDLR expression levels on transduction efficiency showed that higher LDLR expression analysis correlated with higher transduction efficiency (Pearson correlation coefficient $r = 0.6750$, $p < 0.0001$) (Figures 2F and 2G). In addition, we observed that higher virus titers resulted in higher GFP expression levels, but also led to more cell death (Figure S2). Therefore, we determined a MOI of 10 to be the optimal virus concentration for the transduction of NK-92 cells. Statins enhanced lentiviral transduction efficiency of NK-92 cells around 2-fold when treated with atorvastatin, rosuvastatin, or simvastatin. While lentiviral transduction in the presence of statins has a negative impact on cell viability, this effect is dose-dependent, and a 5 μM dose of statins can be used without major negative effects on cell viability.

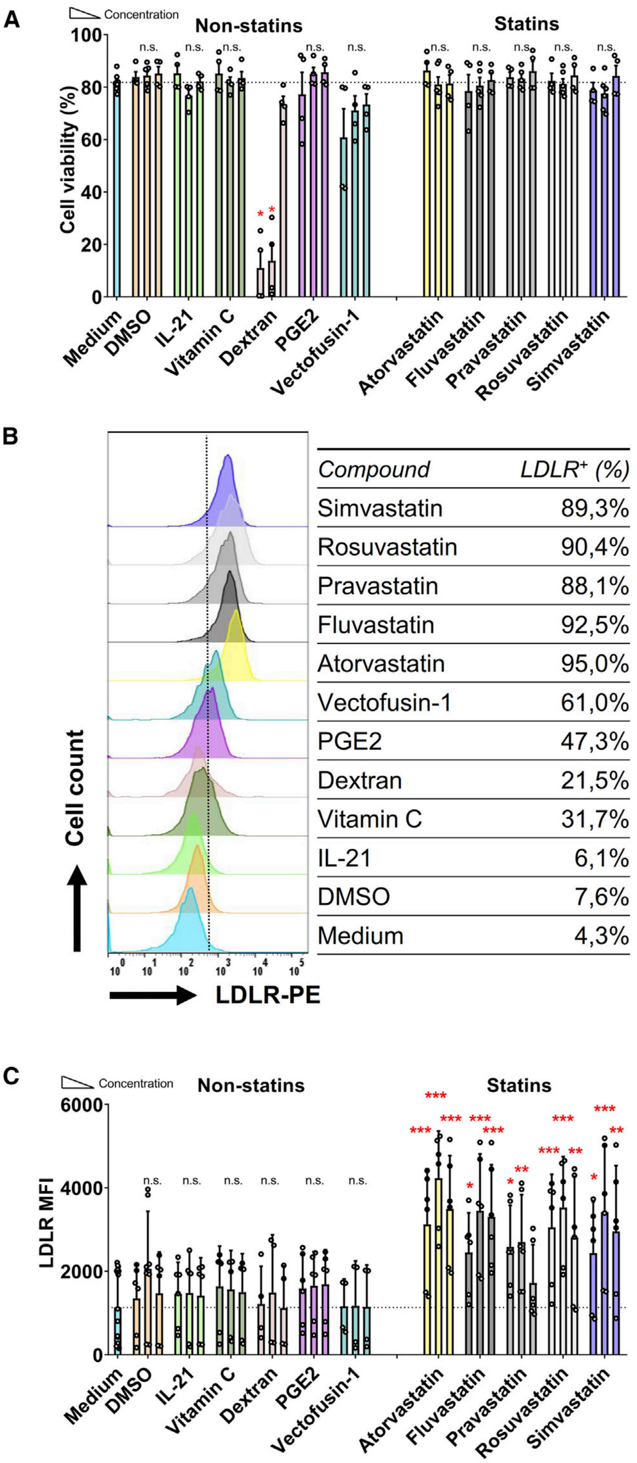

Figure 1. LDL-Receptor Upregulated by Statins on NK-92 Cells

(A) Viability of NK-92 cells after 36 h of co-culture with different concentrations of compounds in MEM medium with 100 U/mL IL-2. Dead cells were stained by the fixable Aqua V500. NK-92 cells were seeded at 0.1 × 106/mL in 96-well plates, 200 μL in each well. IL-21 was added at 20 ng/mL, 5 ng/mL, and 0.5 ng/mL. Vitamin C was used at concentrations of 500 μg/mL, 50 μg/mL, and 5 μg/mL. Dextran was used at 80 μg/mL, 8 μg/mL, and 0.8 μg/mL. PGE2 was used at 100 μM, 10 μM, and 1 μM. Vectofusin-1 was used at 50 μg/mL, 5 μg/mL, and 0.5 μg/mL. All the statins were used at 20 μM, 5 μM, and 0.5 μM. Concentration height is from left to right, indicated with the triangle. DMSO was used as solvent for the statins and was taken along as negative control (B) LDLR expression level of NK-92 cells after 36 h co-culture with the compounds are expressed as median fluorescence intensity (MFI). Overlay histogram of LDLR expression on NK-92 cells under every middle concentration of all compounds are displayed. The table indicates the fraction of LDLR-positive cells. (C) LDLR levels were upregulated by statins, but not by non-statin compounds. Data are shown as mean ± SD; Pooled data from 4 independent experiments performed at different times. Data analysis was performed by a two-way ANOVA and Bonferroni post-tests in comparison to medium. For cell viability, Mann-Whitney U test was used to compare with medium group.

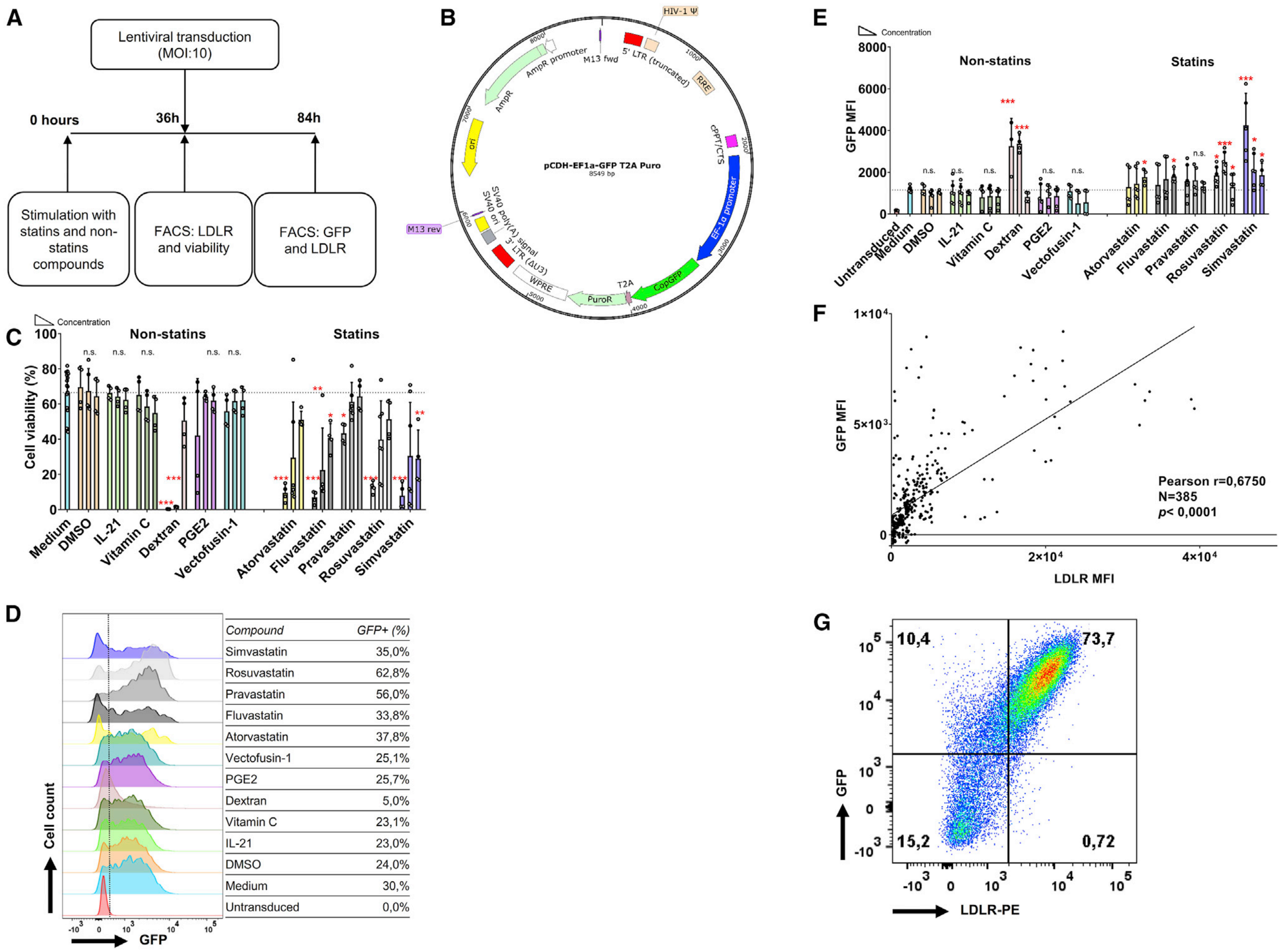

Figure 2. NK-92 Cell Transduction Efficiency Is Enhanced by Statins

(A) Flow chart of procedures using NK-92 cells for transduction with or without compounds. (B) Map of 3rd generation lentiviral vector pCDH-EF1a-GFP-puro. (C) Viability of NK-92 cells 48 h after viral transduction, following culture in the presence of the different compounds at the indicated concentrations. After co-culturing for 36 h, medium was exchanged in each well. Dead cells were detected by fixable Aqua V500. (D) Flow cytometric overlay histograms of GFP expression in NK-92 cells cultured with the middle concentration of each compound. VSV-G lentivirus was added at the MOI of 10:1 in the presence of 10 μg/mL protamine sulfate for transduction. (E) NK cell transduction efficiency was determined by GFP expression in living NK-92 cells. (F) Pearson correlation analysis between LDLR MFI before transduction and 48 h after transduction of NK-92 cells. (G) One representative example of LDLR co-expression with GFP on NK-92 48 h after viral transduction was showed. Data shown as mean ± SD. Pooled data from n = 4 independent experiments performed at different times.

Statin-Induced Reduction in NK Cell Cytotoxicity Is Reversed by GGPP

NK cell immunotherapy builds on the intrinsic cytotoxic capacity of the cells. For NK cells, it has previously been demonstrated that statins can inhibit their cytotoxic capacity.[26,28] Indeed, using the NK-92 cells, we observed almost complete inhibition of cytotoxicity against the K562 chronic myelogenous leukemia (CML) cell line 48 h after lentiviral transduction in the presence of atorvastatin, fluvastatin, or simvastatin, while cytotoxicity was slightly decreased in the presence of rosuvastatin (Figure 3A). However, pravastatin did not alter the cytotoxic capacity of NK-92 cells on K562 cells. Interestingly, geranylgeranyl-pyrophosphate (GGPP), a key molecule in biosynthesis downstream pathways of HMG-CoA reductase,[29] could completely reverse the suppression statin-induced NK cell cytotoxicity (Figure 3B), while maintaining the statin-induced increase in lentiviral transduction (Figure 3C). We also tested whether the cytokine IL-2, that is commonly used to activate NK cells and NK cell lines, could reverse the negative effects of statins on NK cell cytotoxicity, as IL-2 was previously shown to be sufficient to overcome the negative impact of statins on cytotoxicity.[30] However, in NK-92 cells, a high dose of IL-2 (1,000 U/mL) alone was not sufficient to restore their cytotoxic capacity (Figure S3).

To unravel what NK cell cytotoxic pathway was impacted by statins, we analyzed expression of the degranulation marker

CD107a, granzyme B, apoptosis-inducing FAS ligand and interferon-γ (IFN-γ) secretion (Figure S4). Indeed, we observed downregulation of CD107a and granzyme B levels in statin-treated NK-92 cells compared to control cells (Figures S4A and S4B). Importantly, these effects were reversed by GGPP, while GGPP did not influence any of the other analyzed cytotoxicity pathways. Thus, statins can importantly reduce NK-92 cell cytotoxicity, but GGPP can fully reverse this effect completely (Figure 3C).

Statins Upregulate LDLR Expression and Enhance the Viral Transduction Efficiency of Primary Human NK Cells

While lymphoma-derived NK-92 cells have been used for clinical applications,[31] human primary NK cells are a heterogeneous population of cells that in general shows better cytotoxic capacities.[32] We checked freshly isolated human primary NK cells and found that the CD56^{dim} population expressed more LDLR than the CD56^{bright} population (Figures S5A and S5B). Therefore, we next tested whether statins can also be used to enhance lentiviral transduction via LDLR upregulation in primary human NK cells. First, we confirmed that statins (5 μM) and the non-statin compounds did not negatively impact NK cell viability (Figure 4A). The only exception was dextran, which as previously shown had a marked negative impact on NK-92 viability (Figure 1A). In the statin-treated groups, we observed a trend of LDLR upregulation, which was only significantly upregulated 2-fold in the rosuvastatin-treated NK cells (Figures 4B and 4C). Further analysis of LDLR expression showed that most likely only a subset of primary NK cells responds to statin treatment (Figure 4C).

We subsequently transduced primary NK cells with the GFP-expression lentiviral vector that we used before to transduce NK-92 cells (Figure 2A). In contrast to NK-92 cells, primary NK cells can be transduced in the presence of statins or non-statins without significant negative effects on cell viability. However, we did not observe significant differences in viability after treatment with statins (Figure 5A). Meanwhile, we observed a 2- to 3-fold increase in GFP expression levels in statin-treated NK cells compared to the non-statin control group (Figures 5B and 5C). 48 h after transduction, genomic DNA was extracted from the VSV-G transduced primary NK cells and PCR was used to confirm integration of transgene (Figure S6). Indeed, LDLR expression levels positively correlated with GFP expression levels after lentiviral

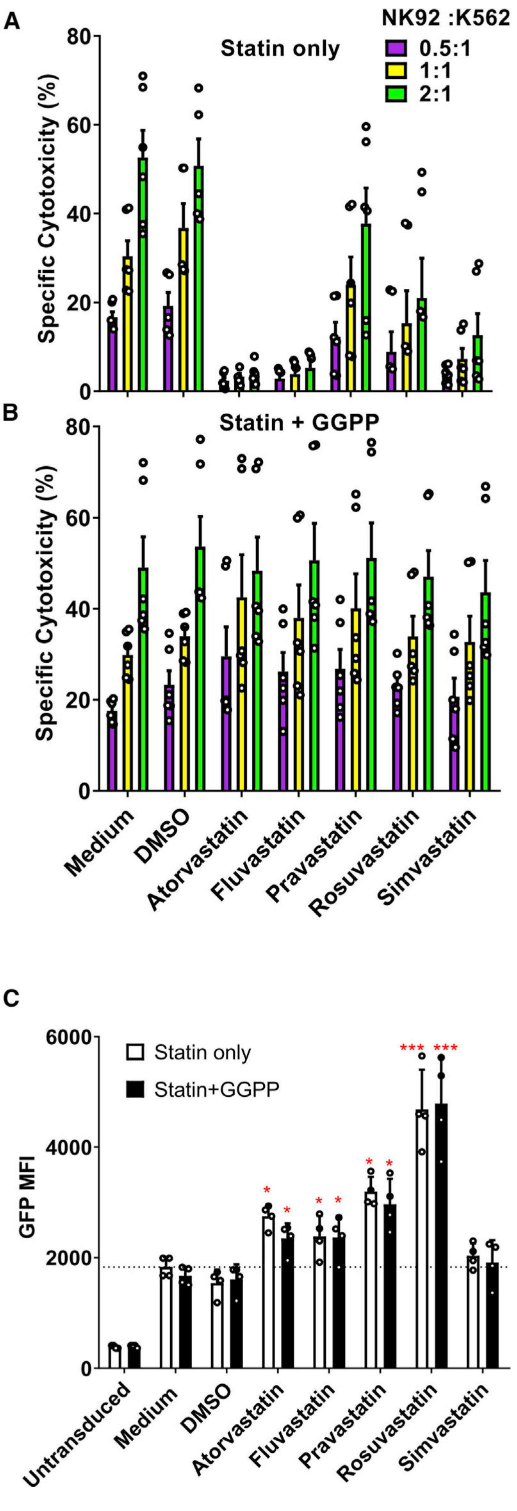

Figure 3. Statins Inhibited NK-92 Cell Cytotoxicity, which Could Reversed by GGPP without Influencing GFP Expression

(A) NK-92 cell cytotoxicity was suppressed by 5 μM lipophilic statins after 36 h incubation. However, NK-92 cells' cytotoxicity was not changed by pravastatin and not 100% restrained by rosuvastatin. NK-92 cells were counted after 36 h exposure to statins and mixed in specified ratios of effector cells to target cells (E/T ratio) in 96-well plates. K562 target cells were seeded at 20,000 cells per well. The cytotoxicity assay was performed during 4 h. Cytotoxicity was quantified as percentage dead target cells using flow cytometry. (B) The inhibition of statins on NK-92 cell cytotoxicity could be reversed by 10 μM GGPP. Data shown is 1 representative experiment out of 3 independent assays. (C) The statin induced enhancement of NK-92 cells transduction efficiency was not altered by GGPP. NK-92 cells were incubated with statins in the presence of GGPP or not during 36 h. Then, culture medium was refreshed and VSV-G at MOI of 10:1 was added. Data were showed as mean ± SD and derived from n = 4 independent experiments performed at different times. Data analysis was performed by pair signed-rank test between statins only group and statins plus GGPP group.

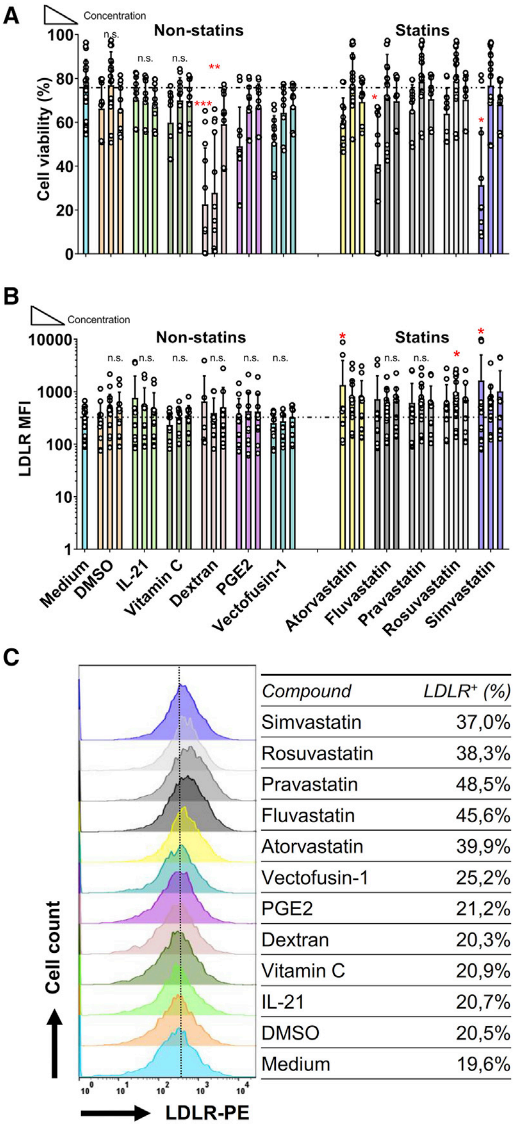

Figure 4. LDL-Receptor Upregulation by Statins on Human Primary NK Cells

(A) Viability of NK cells after 36 h stimulation with different compounds as previous described. Human primary NK cells were co-cultured with compounds for 36 h at a density of 0.1 × 10^6/mL. NK cells were pre-activated overnight in RPMI-1640 with 1,000 U/mL IL-2. (B) The LDLR expression on primary NK cell was upregulated by rosuvastatin, however, in the non-statin groups no significant difference was found when compared to medium. (C) Overlay histogram of LDLR expression on NK cells stimulated 36 h with different compounds. Data are from n = 6 independent experiments performed at different times. Data analysis was performed by a two-way ANOVA and Bonferroni post-tests in comparison to medium. For cell viability, Mann-Whitney U test was used to compare with medium group.

transduction (Figures 5D and 5E; Pearson correlation coefficient $r = 0.6159$, $p < 0.0001$), demonstrating that rosuvastatin that induce LDLR expression can be used to enhance lentiviral transduction.

Rosuvastatin plus GGPP Promotes Lentiviral Transduction through an Increase of LDLR Expression on the Surface of Primary NK Cells

Statins upregulated the LDLR expression (Figure 6A) without negative effects on cell viability (Figure 6B). As for NK-92 cells, higher LDLR expression levels enhanced VSV-G lentiviral transduction in the presence of GGPP (Figure 6C), without concerning the viability before or after transduction on primary NK cells (Figures 6B and 6D). Above all, among 5 kinds of statins, rosuvastatin was the most potent compound to both upregulate the LDLR on NK cells and increase the transduction. The LDLR and GFP were co-expressed in NK-92 (Figure 6E), while primary NK cells have more GFP cells when the LDLR was higher expressed (Figure 6F). After 36 h statins stimulation, the killing ability of primary NK cells in rosuvastatin was decreased. Fortunately, this suppressive effect was completely reversed by GGPP (Figure 7). Thus, a combination of rosuvastatin with GGPP in the culture medium augmented the transduction efficiency of primary NK cells.

DISCUSSION

Achieving high and effective transduction remains a big hurdle for the application of adoptive transfer of genetically modified NK cell therapies in the clinic.[7] Numerous methods have been explored to improve lentiviral transduction efficiency.[33] Here, we tested various statins and showed their enhancing effect on the transduction efficiency on NK cells by increasing the LDLR expression, receptor for VSV-G. The average increased ratio of the LDLR expression by statins both on NK-92 cells and primary human NK cells is approximately 1.5–3 times. The data in this study showed that, compared with the other statins, the lipophilic drug rosuvastatin most potently upregulated LDLR expression on NK cells, resulting in increased transduction efficiency. The addition of GGPP completely reversed the negative impact of statins on NK cell cytotoxicity.

The main biological function of statins is induction of LDLR expression on the cell surface of liver cells through inhibition of hepatic cholesterol synthesis.[34] Upregulation of LDLR results in increased clearance of serum cholesterol. Clinical data shows a significant difference in prevention of coronary artery disease between patients treated with lipophilic or hydrophilic statins.[35,36] The biological function of statins was shown to be dose-dependent with rosuvastatin seemingly having the highest activity.[37] Here, we used statins to increase the LDLR expression to allow VSV-G pseudotyped viruses to enter the NK cell more efficiently. We observed the LDLR expression increasing is time course dependent of statins at 5 μM (Figure S1); however, LDLR is not dose dependent both in NK-92 (Figure 1B) and primary NK cells (Figure 4B). A similar study demonstrated that LDLR expression is not dose dependent.[38] We also found no clear dose-dependent effects of the statins at the different doses tested (0.5 μM, 5 μM, and 20 μM). However, we found that high concentrations of statins (more than 20 μM) induced cell death, possibly due to negative effects on overall cell metabolism, thereby also inhibiting LDLR expression levels. Similarly, lower concentration of statins (<0.5 μM) may not be enough to induce LDLR high expression.

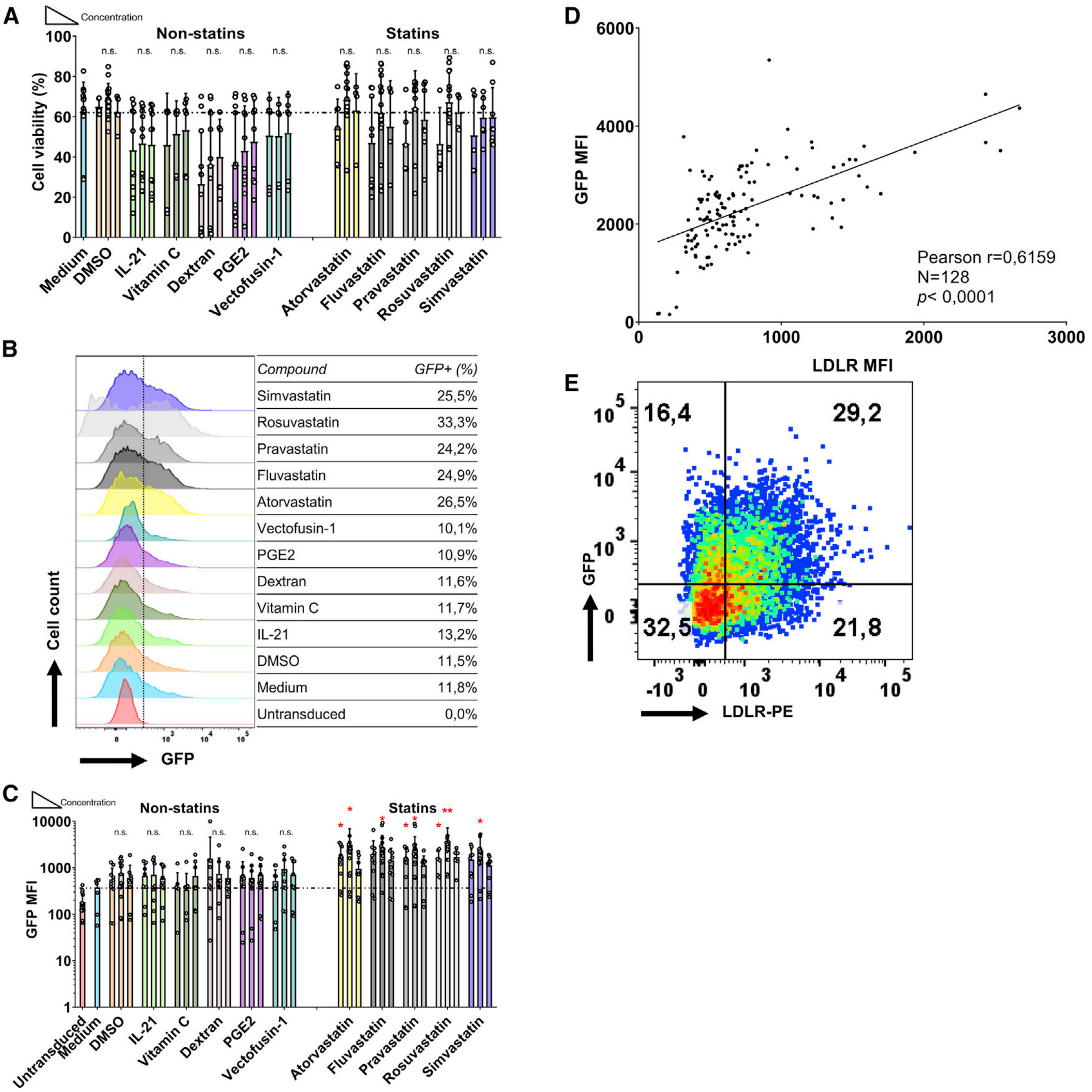

Figure 5. Lentiviral Transduction of Human Primary NK Cells

(A) Viability of primary NK cells 48 h after transduction with different compounds at the indicated concentrations. (B) Flow cytometric overlay histogram of GFP expression in NK cells. VSV-G lentivirus was added at the MOI of 10:1 in the presence of 10 μg/mL protamine sulfate. (C) NK cell transduction efficiency was determined by GFP expression in living NK cells. GFP expression was higher after culture with rosuvastatin when compared with DMSO and medium group. (D) Pearson correlation analysis of LDLR MFI (before transduction) and GFP MFI (48 h after transduction) on NK cells. (E) One representative LDLR co-expression with GFP on NK after 48 h viral transduction. Data was showed as mean ± SD from n = 6 independent experiments performed at different times. Data analysis was performed by a two-way ANOVA and Bonferroni post-tests in comparison to medium. For cell viability, Mann-Whitney U test was used to compare with medium group.

The inhibitory effects on proliferation and cytotoxicity by statins have been reported in several studies.[28,29] When compared with lipophilic statins (e.g., simvastatin and fluvastatin), hydrophilic statins (including pravastatin and rosuvastatin) showed milder or no suppression of proliferation and functional capacity on IL-2 stimulated NK cells (Figure 3).[39] The viability of NK-92 cells in the medium control group before and after transduction decreased from 80% decrease to 70% (Figures 1A and 2C), while in primary NK cells, cell viability decreased from 75% to 65% (Figures 4A and 5A). Before transduction, were found no differences in cell viability in NK-92 cells or primary NK cells between the statin-treated group and the non-statin group. However, after VSV-G lentivirus transduction, the 20 μM of statins group showed negative effects on the viability of both NK-92 cells and primary NK cells. This effect is mainly due to the toxicity of VSV-G protein and protamine sulfate, with an additional detrimental effect of statins on NK cell viability. The inhibitory effects of statins on NK cell cytotoxicity could, among others, be due to the reduction of NK-target (tumor) cell adhesion, granule exocytosis

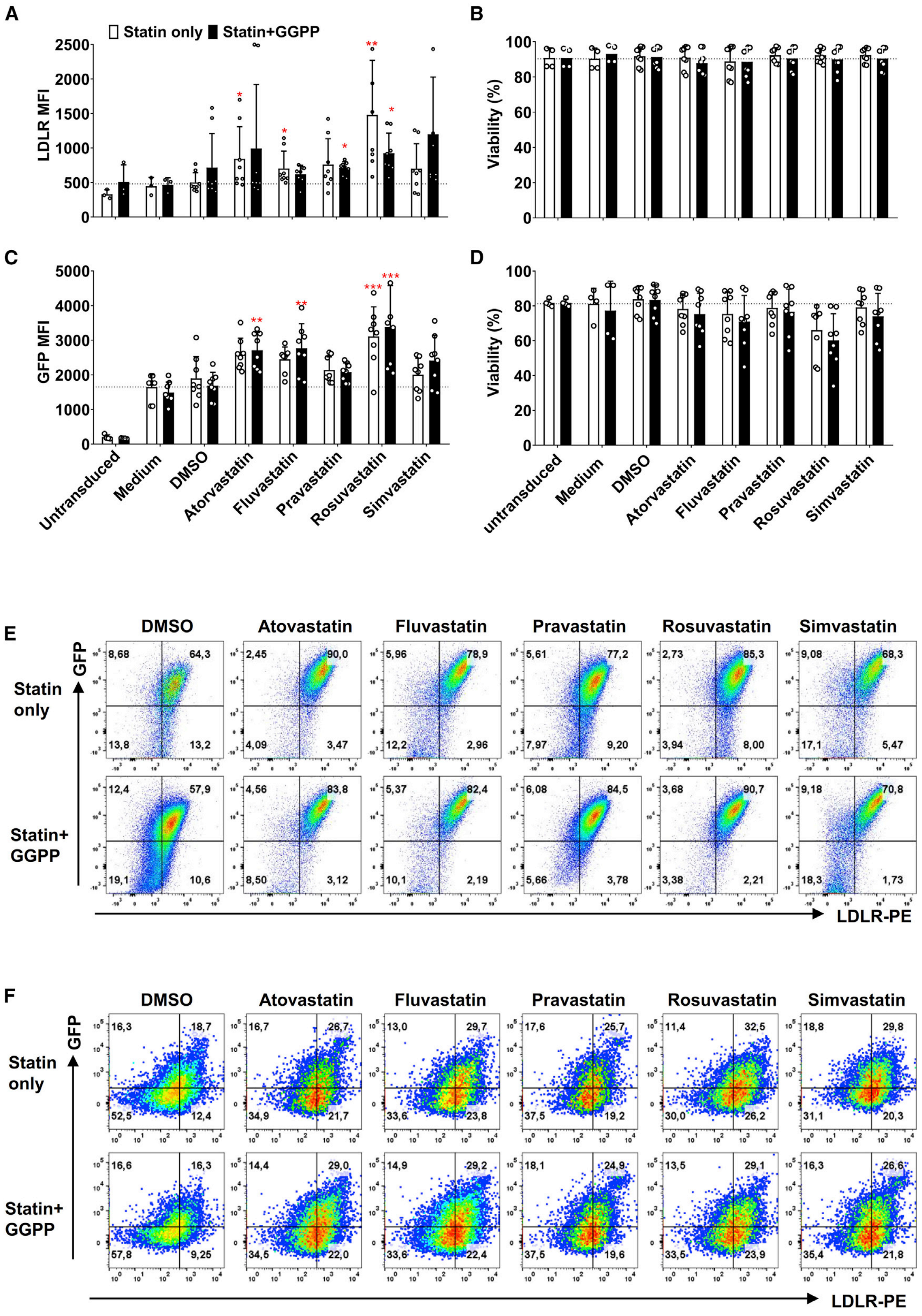

(legend on next page)

(perforin and granzymes release).[26,27] The release of cytotoxic lytic granules from NK cells is strongly dependent on phosphatidylinositol-specific phospholipase Cγ (PLCγ) phosphorylation and intracellular free calcium.[40] Whether statins alter the abundance of intracellular Ca^{2+} in NK cells is debated: Raemer et al.[28] showed that simvastatin had no influence on Ca^{2+} flux in human primary NK cells, while Poggi et al.[27] demonstrated fluvastatin reduced intracellular free Ca^{2+} concentration in human NK cell clones. This discrepancy might be due to the different statins used or NK cell source variation. Furthermore, statins have been reported to change the killing components of NK cells, such as CD95L (FasL) and IFN-γ.[27] In this study, we also investigated the CD107a degranulation, granzyme B, FasL expression, and IFN-γ secretion after co-culture of NK cells with statins (Figure S4). As previous studies[26,27] already demonstrated, CD107a degranulation and FasL levels were reduced upon statin treatment. Interestingly, IFN-γ secretion was not altered. This could be due to cytokines and cell-cell contact with neighboring cells, which has been reported in earlier studies.[30,41] These results could imply that the statin-induced inhibition of NK killing capacity might be due to changes in the degranulation process.

As described in the results section, statins decreased NK cell cytotoxicity. However, this effect could be reversed completely by GGPP.[28] GGPP is synthesized by HMG-CoA reductase and is independent from the cholesterol metabolism. GGPP or mevalonate reversed the inhibitory effects of statins on HMG-CoA reductase.[29] Crosbie et al. showed that GGPP or mevalonate was able to alter the cell cycle and DNA synthesis of NK cells, thus abrogating the negative effects of statins on NK cell proliferation.[29,42]

In addition to statins, other approaches have also been tested to improve high NK cell transduction to create CAR-NK cells.[43] Polycations like polybrene, dextran, poly-L-lysine, and protamine sulfate are supposed to eliminate the charges on cell membranes, thereby enhancing viral transduction efficiency.[15,44] In a direct comparison of polybrene, protamine sulfate, and dextran sulfate,[15] it was shown that dextran-treated NK cells show the highest transduction efficiency at 38% GFP-positive cells, whereas no GFP expression was detected in cells treated with either polybrene or protamine sulfate. In contrast to the present study, their research showed that 8 μg/mL dextran had no influence on the viability and killing capacity of NK cells, while we observed that this concentration of dextran was detrimental for NK cell viability (below 20%; Figure 1A). This difference might be due to the use of freshly isolated NK cells, while Nanbakhsh et al. used expanded NK cells.[15] The second approach to increase higher NK cell transduction efficiency is by cytokine or mitogen stimulation. Previous studies demonstrated that LDLR upregulation, in conjunction with enhanced proliferation and cytotoxicity, can also be achieved by stimulation with IL-2.[45] Soluble IL-2 plus IL-12 stimulation enhances VSV-G lentiviral transduction, which could be further enhanced by 1 μg/mL PHA.[21] Culturing primary human NK cells on K562 feeder cells with expressing membrane-bound IL-21 and 4-1BBL also augmented gene transduction by 50%.[46,47] Third, suppression of intracellular antiviral defense mechanisms are also described to increase lentiviral transduction of NK cells. BX795, which is an inhibitor of the TBK1/IKKe complex that controls antiviral responses, is able to boost lentiviral gene transduction efficiency by 3.8-fold.[12] Next, higher transduction efficiency may be achieved with other pseudotypes of viruses, like alpharetroviral vectors[48] or baboon envelope pseudotyped lentivirus.[47] Finally, methods have also been developed to obtain genetically engineered NK cells from HSC[49] or induced pluripotent stem cells.[50]

Statins also have anti-inflammatory and immunomodulatory properties in clinical treatment.[51] Given that statins facilitate the entry of lentiviruses into cells, this could render patients more susceptible to virus infection. Even though some small studies indicate that statins indeed promoted virus infection or activity (e.g., in herpes zoster[52] and respiratory viral infections[53]), other studies indicate that statins restrict virus activity (e.g., in HIV[54] and Ebola infection[55]), while a third group shows no effect.[53,56] Most retrospective studies have intrinsic methodological limitations and too few relevant randomized controlled trials have been performed on relation between statins and viral infectious incidence.[56] Altogether, at this time there is no body of evidence that allows us to draw definitive conclusions on the effects of clinical statin use on virus infections.

In the current paper, we describe a novel strategy to improve the transduction on human NK cells by increasing the expression of the LDLR. NK cells expressing higher LDLR levels on the surface could get easier transduced with VSV-G lentivirus by helping the VSV lentivirus entry into the NK cell (Figures 2F and 5D). Now the structural composition of how VSV-G recognizes the LDLR on the cell surface has been elucidated,[57] we can imagine that specific overexpression of the cysteine-rich domains (CR2 or CR3) of the LDLR on NK cells could enhance the transduction efficiency. The limitation of the approach is that human primary NK cells express low levels of LDLR. However, other VSV-G receptors may be present on NK cell surface, like the leucine-rich repeat-containing G protein-coupled receptor 4 (Lgr4),[58] HSP90B1,[59] and LDL-receptor other family members.[17]

Figure 6. Statins with GGPP Enhance the Transduction Efficiency of NK-92 and Primary NK Cells

(A) LDL-receptor expression on primary NK cells after 36 h stimulation with 5 μM statins, with or without 10 μM GGPP. Primary NK cells were co-cultured with 5 μM statins in the presence or absence of GGPP at a density of 0.1 × 106/mL (B) Viability of primary NK cells after 36 h cultured in the presence of 5 μM statins with or without 10 μM GGPP. (C) GFP expression in primary NK cells after lentiviral transduction. After statins incubation, the culture medium was refreshed and VSV-G added at MOI of 10:1. (D) Viability of primary NK cells after 48 h transduction. (E and F) Representative flow cytometry LDLR and GFP co-expression in NK-92 (E) and primary NK cells (F) after 48 h lentiviral transduction and culturing with statins with or without GGPP. Data is showed as mean ± SD from n = 4 independent experiments performed at different times. Data analysis was performed by a two-way ANOVA and Bonferroni post-tests in comparison to medium. For cell viability, Mann-Whitney U test was used to compare with medium group.

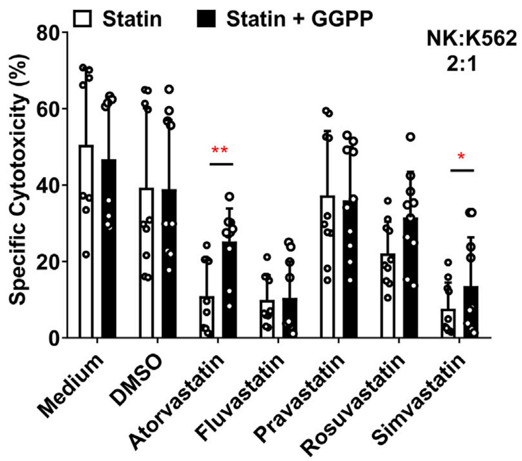

Figure 7. Cytotoxicity of Primary NK cells Is Inhibited by Statins, but Restored by GGPP

Human primary NK cell cytotoxicity was suppressed by lipophilic statins. NK cell cytotoxicity was not changed by pravastatin and restrained by rosuvastatin. NK cells were incubated with statins in presence of GGPP or not. NK cells were seeded at the density of 0.1 × 10^6/mL in 96-well plates with or without statins. After removing the supernatant, NK cells were added in a 2:1 E:T ratio: 40,000 cells NK cells versus 20,000 K562 cells per well. Cytotoxicity assay was performed for 4 h. The inhibition of statins on primary NK cell cytotoxicity could only be completely reversed by addition of 10 μM GGPP after rosuvastatin stimulation. Data was showed from 4 independent assays. Data analysis was performed by pair signed-rank test between statins only group and statins plus GGPP group.

For future therapeutic applications, rosuvastatin plus GGPP currently is the most potent combination that increases VSV-G lentivirus transduction efficiency without a reduction of NK cell cytotoxicity. This finding is important for the both scientists and clinicians, as it facilitates the transduction of NK cells that are known to be hard to transduce, but it holds important promise for cancer adoptive cell therapy.

MATERIALS AND METHODS

Cell Lines and Culture

NK-92 cells (CRL-2407, ATCC, Manassas, VA, USA) were cultured in alpha Minimum Essential medium (Thermo Fisher Scientific, Waltham, MA, USA) without ribonucleosides and deoxyribonucleosides, supplemented with 2 mM L-glutamine, 2.2 g sodium bicarbonate (Sigma-Aldrich, Munich, Germany), 0.2 mM inositol (Sigma-Aldrich), 0.1 mM 2-mercaptoethanol (Sigma-Aldrich), 0.02 mM folic acid (Sigma-Aldrich), 100 U/mL recombinant IL-2 (Proleukin, Novartis, Basel, Switzerland), 12.5% horse serum (ATCC), 12.5% fetal calf serum (FCS, Greiner Bio-One, Frickenhausen, Germany), and 1% penicillin/ streptomycin (Thermo Fisher Scientific). K562 (ATCC CCL-243) cells were cultured in IMDM supplemented with 10% FCS (Greiner Bio-One) and 1% penicillin/streptomycin (Thermo Fisher Scientific). 293FT cells (R700-07, Thermo Fisher Scientific) were cultured in DMEM/high glucose medium supplement with 10% FCS (Greiner Bio-One), 0.1 mM MEM non-essential amino acids (NEAA, Thermo Fisher Scientific), 2 mM L-glutamine (Thermo Fisher Scientific), and 1 mM MEM sodium pyruvate (Thermo Fisher Scientific). The Jurkat cell line (ACC 282, DSMZ, Braunschweig, Germany) was cultured in RPMI-1640 medium (Thermo Fisher Scientific) with 10% FCS and 1% penicillin/streptomycin.

Human NK Cell Isolation and Activation

Primary human NK cells were isolated from anonymous buffy coats (Sanquin, Maastricht, the Netherlands). The use of buffy coats, being a byproduct of a required Medical Ethical Review Committee (METC) procedure, does not need ethical approval in the Netherlands under the Dutch Code for Proper Secondary Use of Human Tissue. NK cells were subsequently isolated by negative selection with an NK cell isolation kit (130-092-657, Miltenyi Bbiotec, Bergisch Gladbach, Germany) using MACS beads as previously described.[60] For short-term activation, NK cells were cultured in RPMI-1640 medium (GIBCO) supplemented with 10% fetal calf serum (Greiner Bio-One) and 100 U/mL penicillin-streptomycin (GIBCO). NK cells were activated overnight with 1,000 IU/mL recombinant human IL-2. All cells were cultured with 5% CO_2 at 37°C in a humidified cell culture Sanyo MCO-20AIC incubator (Sanyo Electric, Osaka, Japan).

Co-culture of NK Cells with Statins and Non-Statin Compounds

NK-92 cells were seeded at 0.1 × 10^6 cells/mL in round-bottom 96-well plates (3799, Corning Life Sciences B.V., Amsterdam, the Netherlands). Atorvastatin (10493, Cayman Chemical), fluvastatin (10010337, Cayman Chemical), pravastatin (10010342, Cayman Chemical), rosuvastatin (12029, Cayman Chemical), and simvastatin (10010344, Cayman Chemical) were dissolved in DMSO (Sigma-Aldrich). Statins were used at final concentration of 20 μM, 5 μM, and 0.5 μM. DMSO was diluted in the same volume and served as solvent control. IL-2, IL-21 (Thermo Fisher Scientific), vitamin C (Sigma-Aldrich), dextran (Sigma-Aldrich), prostaglandin E2 (PGE2, Sigma-Aldrich), protamine sulfate (Sigma-Aldrich), and vectofusin-1 (Miltenyi Biotec) were dissolved in distilled water (GIBCO). DMSO was added 0.8 μL, 0.2 μL, and 0.02 μL, respectively, at the same volume as the statin group in 96-well plates. IL-21 was added at 20 ng/mL, 5 ng/mL, and 0.5 ng/mL. Vitamin C was used at concentrations of 500 μg/mL, 50 μg/mL, and 5 μg/mL as described previously.[23] Dextran was used at 80 μg/mL, 8 μg/mL, and 0.8 μg/mL.[15] PGE2 was used at 100 μM, 10 μM, and 1 μM.[22] Vectofusin-1 was used at 50 μg/mL, 5 μg/mL, and 0.5 μg/mL.[14,61] GGPP ammonium salt was purchased from Sigma-Aldrich and was added at 10 μM in co-culture assays.[29]

Vectors and Lentivirus Production and NK Cells Transduction

All the virus procedures were under the surveillance by the Center for Research Innovation, Support and Policy (CRISP) of Maastricht University Medical Center (Genetic Modification License number: GGO-00-177). pCDH-EF1-copGFP-T2A-Puro was a gift from Kazuhiro Oka (Addgene plasmid # 72263, Watertown, MA, USA). pRSV-Rev (Addgene # 12253), pMDLg/ pRRE (Addgene # 12251), and pMD2.G (Addgene # 12259) were gifts from Didier Trono.[62] Plasmids were expanded in Stbl3 *E. coli* (Thermo Fisher Scientific) and prepared by followed the instruction of NucleoBond Xtra Midi Kit (Macherey-Nagel GmbH, Düren, Germany). All these four plasmids were mixed with the ratio 5:2:2:1 in 40 μg and were transfected in one

150 mm cell culture dish of 293FT cells in the presence of 80 μg polyethylenimine (PEI, Polysciences, Warrington, PA, USA). After 48 h, every 24 h and continually for 5 days, virus supernatant was collected and filtered through a 0.45 μm syringe filter (Merck Millipore, Burlington, MA, USA). Pooled virus supernatant was concentrated using Lenti-X Concentrator (Takara, Saint-Germain-en-Laye, France) according to the manufacturer's protocol. Viral titers were determined using Jurkat cells by performing 2 × serial dilutions and detection of GFP expression using flow cytometry 48 h post-transduction. VSV-G lentiviruses were added to cells at a MOI 10 in the presence of 10 μg/mL protamine sulfate after 36 h of statin co-incubation.

Flow Cytometry and Antibodies Staining

For flow cytometric analysis, cells were stained with Live/Dead Fixable Aqua Dead Cell Stain Kit (Thermo Fisher Scientific) in PBS on ice for 30 min. Then cells were stained using the following antibodies recognizing human antigens: CD3 (Clone OKT3, BD Biosciences, San Jose, CA, USA), CD56 (Clone REA196, Miltenyi Biotec), LDLR (Clone C7, BD Biosciences), CD95L (Clone NOK-1, BD Biosciences), CD107a (Clone H4A3, Miltenyi Biotec), granzyme B (Clone REA226, Miltenyi Biotec), and IFN-γ (REA600, Miltenyi Biotec). Antibodies mixture in fluorescence-activated cell sorting (FACS) staining buffer (PBS + 1% FCS) were added to NK cells and incubated at 4°C for 30 min. Fluorescence was read on a BD FACS CantoII flow cytometer. Data were analyzed with FlowJo 10.1 (TreeStar, Ashland, OR, USA) software.

Cytotoxicity Assay

The NK cell killing ability against tumor cells was determined in a 4 h flow cytometry-based assay. Tumor cells were pre-labeled with Cell Tracker Deep Red Dye according to the manufacturer's protocol (Thermo Fisher Scientific) and were cultured overnight. Tumor cells were harvested and seeded at 20,000 cells per well in round-bottom 96-well plates. NK cells were seeded in duplicates with different effector to target ratios (E/T ratio) and cultured in RPMI-1640 medium for 4 h. After 4 h, dead Deep Red-labeled tumor cells were measured with Live/Dead Fixable Aqua Dead Cell Stain Kit (Thermo Fisher Scientific) by flow cytometry. Specific cytotoxicity was calculated as previously described.[60]

Statistical Analysis

All statistical tests used in this study were completed with GraphPad Prism 8 software (Graphpad Software, San Diego, CA, USA). The specific statistical tests used for each comparison are specifically annotated in the figure legends, respectively. For performing multiple comparisons, we used two-way ANOVA and Bonferroni post-tests comparing to the medium control group. For cell viability, Mann-Whitney U test was used to compare with the medium control group. $^{*}p < 0.05$, $^{**}p < 0.01$, and $^{***}p < 0.001$. Results were considered non-significantly (n.s.) different if $p \geq 0.05$.

SUPPLEMENTAL INFORMATION

Supplemental Information can be found online at https://doi.org/10.1016/j.omtm.2020.03.017.

AUTHOR CONTRIBUTIONS

Y.G., R.G.J.K.W., and I.J. designed and performed experiments. R.G.J.K.W. and W.T.V.G. evaluated data. A.J.G. contributed protocols and reagents. Y.G., R.G.J.K.W., and W.T.V.G. wrote the manuscript. G.M.J.B. and W.T.V.G. conceived and designed the study, supervised the work, and wrote the manuscript. All authors read and approved the final manuscript.

CONFLICTS OF INTEREST

G.M.J.B. and W.T.V.G. are the founders of CiMaas BV. All other authors declare no competing interests.

ACKNOWLEDGMENTS

Y.G. thanks the China Scholarship Council (CSC, grant number 201707720056) for fellowship support. R.G.J.K.W. is supported by a Kootstra Talent Fellowship (Maastricht University) and a Marie Skłodowska-Curie Individual fellowship (799810-TOPNIN; European Union). The research was supported by Cancer Research Foundation Limburg and GROW School of Maastricht University.

REFERENCES

1. Dunbar, C.E., High, K.A., Joung, J.K., Kohn, D.B., Ozawa, K., and Sadelain, M. (2018). Gene therapy comes of age. Science *359*, eaan4672.
2. Zhang, C., Oberoi, P., Oelsner, S., Waldmann, A., Lindner, A., Tonn, T., and Wels, W.S. (2017). Chimeric Antigen Receptor-Engineered NK-92 Cells: An Off-the-Shelf Cellular Therapeutic for Targeted Elimination of Cancer Cells and Induction of Protective Antitumor Immunity. Front. Immunol. *8*, 533.
3. Screpanti, V., Wallin, R.P., Grandien, A., and Ljunggren, H.G. (2005). Impact of FASL-induced apoptosis in the elimination of tumor cells by NK cells. Mol. Immunol. *42*, 495–499.
4. Miller, J.S., Soignier, Y., Panoskaltsis-Mortari, A., McNearney, S.A., Yun, G.H., Fautsch, S.K., McKenna, D., Le, C., Defor, T.E., Burns, L.J., et al. (2005). Successful adoptive transfer and in vivo expansion of human haploidentical NK cells in patients with cancer. Blood *105*, 3051–3057.
5. Liu, E., Marin, D., Banerjee, P., Macapinlac, H.A., Thompson, P., Basar, R., Nassif Kerbauy, L., Overman, B., Thall, P., Kaplan, M., et al. (2020). Use of CAR-Transduced Natural Killer Cells in CD19-Positive Lymphoid Tumors. N. Engl. J. Med. *382*, 545–553.
6. Suen, W.C.-W., Lee, W.Y.-W., Leung, K.-T., Pan, X.-H., and Li, G. (2018). Natural Killer Cell-Based Cancer Immunotherapy: A Review on 10 Years Completed Clinical Trials. Cancer Invest. *36*, 431–457.
7. Carlsten, M., and Childs, R.W. (2015). Genetic Manipulation of NK Cells for Cancer Immunotherapy: Techniques and Clinical Implications. Front. Immunol. *6*, 266.
8. Nguyen, D.N., Roth, T.L., Li, P.J., Chen, P.A., Apathy, R., Mamedov, M.R., Vo, L.T., Tobin, V.R., Goodman, D., Shifrut, E., et al. (2020). Polymer-stabilized Cas9 nanoparticles and modified repair templates increase genome editing efficiency. Nat. Biotechnol. *38*, 44–49.
9. Yang, Y.W., and Hsieh, Y.C. (2001). Protamine sulfate enhances the transduction efficiency of recombinant adeno-associated virus-mediated gene delivery. Pharm. Res. *18*, 922–927.
10. Petrillo, C., Cesana, D., Piras, F., Bartolaccini, S., Naldini, L., Montini, E., and Kajaste-Rudnitski, A. (2015). Cyclosporin a and rapamycin relieve distinct lentiviral restriction blocks in hematopoietic stem and progenitor cells. Mol. Ther. *23*, 352–362.
11. Wang, C.X., Sather, B.D., Wang, X., Adair, J., Khan, I., Singh, S., Lang, S., Adams, A., Curinga, G., Kiem, H.P., et al. (2014). Rapamycin relieves lentiviral vector transduction resistance in human and mouse hematopoietic stem cells. Blood *124*, 913–923.
12. Sutlu, T., Nyström, S., Gilljam, M., Stellan, B., Applequist, S.E., and Alici, E. (2012). Inhibition of intracellular antiviral defense mechanisms augments lentiviral

transduction of human natural killer cells: implications for gene therapy. Hum. Gene Ther. *23*, 1090–1100.

13. Radek, C., Bernadin, O., Drechsel, K., Cordes, N., Pfeifer, R., Sträßer, P., Mormin, M., Gutierrez-Guerrero, A., Cosset, F.L., Kaiser, A.D., et al. (2019). Vectofusin-1 improves transduction of primary human cells with diverse retroviral and lentiviral pseudotypes, enabling robust, automated closed-system manufacturing. Hum. Gene Ther. *30*, 1477–1493.
14. Piovan, C., Marin, V., Scavullo, C., Corna, S., Giuliani, E., Bossi, S., Galy, A., Fenard, D., Bordignon, C., Rizzardi, G.P., and Bovolenta, C. (2017). Vectofusin-1 Promotes RD114-TR-Pseudotyped Lentiviral Vector Transduction of Human HSPCs and T Lymphocytes. Mol. Ther. Methods Clin. Dev. *5*, 22–30.
15. Nanbakhsh, A., Best, B., Riese, M., Rao, S., Wang, L., Medin, J., Thakar, M.S., and Malarkannan, S. (2018). Dextran Enhances the Lentiviral Transduction Efficiency of Murine and Human Primary NK Cells. J. Vis. Exp. *131*, 55063.
16. Burns, J.C., Friedmann, T., Driever, W., Burrascano, M., and Yee, J.K. (1993). Vesicular stomatitis virus G glycoprotein pseudotyped retroviral vectors: concentration to very high titer and efficient gene transfer into mammalian and nonmammalian cells. Proc. Natl. Acad. Sci. USA *90*, 8033–8037.
17. Finkelshtein, D., Werman, A., Novick, D., Barak, S., and Rubinstein, M. (2013). LDL receptor and its family members serve as the cellular receptors for vesicular stomatitis virus. Proc. Natl. Acad. Sci. USA *110*, 7306–7311.
18. Amirache, F., Lévy, C., Costa, C., Mangeot, P.-E., Torbett, B.E., Wang, C.X., Nègre, D., Cosset, F.L., and Verhoeyen, E. (2014). Mystery solved: VSV-G-LVs do not allow efficient gene transfer into unstimulated T cells, B cells, and HSCs because they lack the LDL receptor. Blood *123*, 1422–1424.
19. Cerrato, F., Fernández-Suárez, M.E., Alonso, R., Alonso, M., Vázquez, C., Pastor, O., Mata, P., Lasunción, M.A., and Gómez-Coronado, D. (2015). Clinically used selective oestrogen receptor modulators increase LDL receptor activity in primary human lymphocytes. Br. J. Pharmacol. *172*, 1379–1394.
20. Oelsner, S., Friede, M.E., Zhang, C., Wagner, J., Badura, S., Bader, P., Ullrich, E., Ottmann, O.G., Klingemann, H., Tonn, T., and Wels, W.S. (2017). Continuously expanding CAR NK-92 cells display selective cytotoxicity against B-cell leukemia and lymphoma. Cytotherapy *19*, 235–249.
21. Micucci, F., Zingoni, A., Piccoli, M., Frati, L., Santoni, A., and Galandrini, R. (2006). High-efficient lentiviral vector-mediated gene transfer into primary human NK cells. Exp. Hematol. *34*, 1344–1352.
22. Heffner, G.C., Bonner, M., Christiansen, L., Pierciey, F.J., Campbell, D., Smurnyy, Y., Zhang, W., Hamel, A., Shaw, S., Lewis, G., et al. (2018). Prostaglandin E_2 Increases Lentiviral Vector Transduction Efficiency of Adult Human Hematopoietic Stem and Progenitor Cells. Mol. Ther. *26*, 320–328.
23. Huijskens, M.J., Walczak, M., Sarkar, S., Atrafi, F., Senden-Gijsbers, B.L., Tilanus, M.G.J., Bos, G.M., Wieten, L., and Germeraad, W.T. (2015). Ascorbic acid promotes proliferation of natural killer cell populations in culture systems applicable for natural killer cell therapy. Cytotherapy *17*, 613–620.
24. Parihar, S.P., Guler, R., and Brombacher, F. (2019). Statins: a viable candidate for host-directed therapy against infectious diseases. Nat. Rev. Immunol. *19*, 104–117.
25. Pocathikorn, A., Taylor, R.R., and Mamotte, C.D. (2010). Atorvastatin increases expression of low-density lipoprotein receptor mRNA in human circulating mononuclear cells. Clin. Exp. Pharmacol. Physiol. *37*, 471–476.
26. Hillyard, D.Z., Nutt, C.D., Thomson, J., McDonald, K.J., Wan, R.K., Cameron, A.J., Mark, P.B., and Jardine, A.G. (2007). Statins inhibit NK cell cytotoxicity by membrane raft depletion rather than inhibition of isoprenylation. Atherosclerosis *191*, 319–325.
27. Poggi, A., Boero, S., Musso, A., and Zocchi, M.R. (2013). Selective role of mevalonate pathway in regulating perforin but not FasL and TNFalpha release in human Natural Killer cells. PLoS ONE *8*, e62932.
28. Raemer, P.C., Kohl, K., and Watzl, C. (2009). Statins inhibit NK-cell cytotoxicity by interfering with LFA-1-mediated conjugate formation. Eur. J. Immunol. *39*, 1456–1465.
29. Crosbie, J., Magnussen, M., Dornbier, R., Iannone, A., and Steele, T.A. (2013). Statins inhibit proliferation and cytotoxicity of a human leukemic natural killer cell line. Biomark. Res. *1*, 33.
30. Gruenbacher, G., Gander, H., Nussbaumer, O., Nussbaumer, W., Rahm, A., and Thurnher, M. (2010). IL-2 costimulation enables statin-mediated activation of human NK cells, preferentially through a mechanism involving CD56+ dendritic cells. Cancer Res. *70*, 9611–9620.
31. Klingemann, H., Boissel, L., and Toneguzzo, F. (2016). Natural Killer Cells for Immunotherapy - Advantages of the NK-92 Cell Line over Blood NK Cells. Front. Immunol. *7*, 91–91.
32. Yang, L., Shen, M., Xu, L.J., Yang, X., Tsai, Y., Keng, P.C., Chen, Y., and Lee, S.O. (2017). Enhancing NK cell-mediated cytotoxicity to cisplatin-resistant lung cancer cells via MEK/Erk signaling inhibition. Sci. Rep. *7*, 7958.
33. Johnston, J.M., Denning, G., Moot, R., Whitehead, D., Shields, J., Le Doux, J.M., Doering, C.B., and Spencer, H.T. (2014). High-throughput screening identifies compounds that enhance lentiviral transduction. Gene Ther. *21*, 1008–1020.
34. Oesterle, A., Laufs, U., and Liao, J.K. (2017). Pleiotropic Effects of Statins on the Cardiovascular System. Circ. Res. *120*, 229–243.
35. Izawa, A., Kashima, Y., Miura, T., Ebisawa, S., Kitabayashi, H., Yamamoto, H., Sakurai, S., Kagoshima, M., Tomita, T., Miyashita, Y., et al.; ALPS-AMI Investigators (2015). Assessment of lipophilic vs. hydrophilic statin therapy in acute myocardial infarction – ALPS-AMI study. Circ. J. *79*, 161–168.
36. Bytyçi, I., Bajraktari, G., Bhatt, D.L., Morgan, C.J., Ahmed, A., Aronow, W.S., and Banach, M.; Lipid and Blood Pressure Meta-analysis Collaboration (LBPMC) Group (2017). Hydrophilic vs lipophilic statins in coronary artery disease: A meta-analysis of randomized controlled trials. J. Clin. Lipidol. *11*, 624–637.
37. Law, M.R., Wald, N.J., and Rudnicka, A.R. (2003). Quantifying effect of statins on low density lipoprotein cholesterol, ischaemic heart disease, and stroke: systematic review and meta-analysis. BMJ *326*, 1423.
38. Chan, P.-C., Lafrenière, R., and Parsons, H.G. (1997). Lovastatin increases surface low density lipoprotein receptor expression by retarding the receptor internalization rate in proliferating lymphocytes. Biochem. Biophys. Res. Commun. *235*, 117–122.
39. Tanaka, T., Porter, C.M., Horvath-Arcidiacono, J.A., and Bloom, E.T. (2007). Lipophilic statins suppress cytotoxicity by freshly isolated natural killer cells through modulation of granule exocytosis. Int. Immunol. *19*, 163–173.
40. Bryceson, Y.T., March, M.E., Ljunggren, H.-G., and Long, E.O. (2006). Activation, coactivation, and costimulation of resting human natural killer cells. Immunol. Rev. *214*, 73–91.
41. Janakiram, N.B., Mohammed, A., Bryant, T., Zhang, Y., Brewer, M., Duff, A., Biddick, L., Singh, A., Lightfoot, S., Steele, V.E., and Rao, C.V. (2016). Potentiating NK cell activity by combination of Rosuvastatin and Difluoromethylornithine for effective chemopreventive efficacy against Colon Cancer. Sci. Rep. *6*, 37046.
42. Tatsuno, I., Tanaka, T., Oeda, T., Yasuda, T., Kitagawa, M., Saito, Y., and Hirai, A. (1997). Geranylgeranylpyrophosphate, a metabolite of mevalonate, regulates the cell cycle progression and DNA synthesis in human lymphocytes. Biochem. Biophys. Res. Commun. *241*, 376–382.
43. Bollino, D., and Webb, T.J. (2017). Chimeric antigen receptor-engineered natural killer and natural killer T cells for cancer immunotherapy. Transl. Res. *187*, 32–43.
44. Davis, H.E., Rosinski, M., Morgan, J.R., and Yarmush, M.L. (2004). Charged polymers modulate retrovirus transduction via membrane charge neutralization and virus aggregation. Biophys. J. *86*, 1234–1242.
45. De Sanctis, J.B., Blanca, I., Radzioch, D., and Bianco, N.E. (1996). Expression and function of low-density lipoprotein receptors in CD3-CD16+CD56+ cells: effect of interleukin 2. Cell. Immunol. *167*, 18–29.
46. Streltsova, M.A., Barsov, E., Erokhina, S.A., and Kovalenko, E.I. (2017). Retroviral gene transfer into primary human NK cells activated by IL-2 and K562 feeder cells expressing membrane-bound IL-21. J. Immunol. Methods *450*, 90–94.
47. Colamartino, A.B.L., Lemieux, W., Bifsha, P., Nicoletti, S., Chakravarti, N., Remon, J.S., et al. (2019). Efficient and robust NK-Cell transduction with Baboon Envelope pseudotyped lentivector: a major tool for immunotherapy. bioRxiv *10*, 625285.
48. Suerth, J.D., Morgan, M.A., Kloess, S., Heckl, D., Neudörfl, C., Falk, C.S., Koehl, U., and Schambach, A. (2016). Efficient generation of gene-modified human natural killer cells via alpharetroviral vectors. J. Mol. Med. (Berl.) *94*, 83–93.

49. Lowe, E., Truscott, L.C., and De Oliveira, S.N. (2016). In Vitro Generation of Human NK Cells Expressing Chimeric Antigen Receptor Through Differentiation of Gene-Modified Hematopoietic Stem Cells. Methods Mol. Biol. *1441*, 241–251.

50. Li, Y., Hermanson, D.L., Moriarity, B.S., and Kaufman, D.S. (2018). Human iPSC-Derived Natural Killer Cells Engineered with Chimeric Antigen Receptors Enhance Anti-tumor Activity. Cell Stem Cell *23*, 181–192.e5.

51. Arefieva, T.I., Filatova, A.Y., Potekhina, A.V., and Shchinova, A.M.J.B. (2018). Immunotropic Effects and Proposed Mechanism of Action for 3-Hydroxy-3-methylglutaryl-coenzyme A Reductase Inhibitors Statins 83, 874–889.

52. Chen, H.H., Lin, C.L., Yeh, C.J., Yeh, S.Y., and Kao, C.H. (2015). Statins can increase the risk of herpes zoster infection in Asia. Eur J Clin Microbiol Infect Dis. *34*, 1451–1458.

53. Seo, S., Boeckh, M., Storer, B.E., Schubert, M.M., Rotta, M., Sandmaier, B.M., and Mielcarek, M. (2015). The association between donor and recipient statin use and infections after allogeneic hematopoietic cell transplantation. Bone Marrow Transplant. *50*, 444–448.

54. del Real, G., Jiménez-Baranda, S., Mira, E., Lacalle, R.A., Lucas, P., Gómez-Moutón, C., Alegret, M., Peña, J.M., Rodríguez-Zapata, M., Alvarez-Mon, M., et al. (2004). Statins inhibit HIV-1 infection by down-regulating Rho activity. J. Exp. Med. *200*, 541–547.

55. Shrivastava-Ranjan, P., Flint, M., Bergeron, É., McElroy, A.K., Chatterjee, P., Albariño, C.G., Nichol, S.T., and Spiropoulou, C.F. (2018). Statins Suppress Ebola Virus Infectivity by Interfering with Glycoprotein Processing. MBio *9*, e00660–e00618.

56. Falagas, M.E., Makris, G.C., Matthaiou, D.K., and Rafailidis, P.I. (2008). Statins for infection and sepsis: a systematic review of the clinical evidence. J. Antimicrob. Chemother. *61*, 774–785.

57. Nikolic, J., Belot, L., Raux, H., Legrand, P., Gaudin, Y., and A Albertini, A. (2018). Structural basis for the recognition of LDL-receptor family members by VSV glycoprotein. Nat. Commun. *9*, 1029.

58. Zhang, N., Huang, H., Tan, B., Wei, Y., Xiong, Q., Yan, Y., Hou, L., Wu, N., Siwko, S., Cimarelli, A., et al. (2017). Leucine-rich repeat-containing G protein-coupled receptor 4 facilitates vesicular stomatitis virus infection by binding vesicular stomatitis virus glycoprotein. J. Biol. Chem. *292*, 16527–16538.

59. Hastie, E., Cataldi, M., Marriott, I., and Grdzelishvili, V.Z. (2013). Understanding and altering cell tropism of vesicular stomatitis virus. Virus Res. *176*, 16–32.

60. Mahaweni, N.M., Bos, G.M.J., Mitsiades, C.S., Tilanus, M.G.J., and Wieten, L. (2018). Daratumumab augments alloreactive natural killer cell cytotoxicity towards CD38+ multiple myeloma cell lines in a biochemical context mimicking tumour microenvironment conditions. Cancer Immunol. Immunother. *67*, 861–872.

61. Jamali, A., Kapitza, L., Schaser, T., Johnston, I.C.D., Buchholz, C.J., and Hartmann, J. (2019). Highly Efficient and Selective CAR-Gene Transfer Using CD4- and CD8-Targeted Lentiviral Vectors. Mol. Ther. Methods Clin. Dev. *13*, 371–379.

62. Dull, T., Zufferey, R., Kelly, M., Mandel, R.J., Nguyen, M., Trono, D., and Naldini, L. (1998). A third-generation lentivirus vector with a conditional packaging system. J. Virol. *72*, 8463–8471.

OMTM, Volume *17*

Supplemental Information

Rosuvastatin Enhances VSV-G Lentiviral Transduction of NK Cells via Upregulation of the Low-Density Lipoprotein Receptor

Ying Gong, Roel G.J. Klein Wolterink, Ian Janssen, Arjan J. Groot, Gerard M.J. Bos, and Wilfred T.V. Germeraad

Supplemental Figures

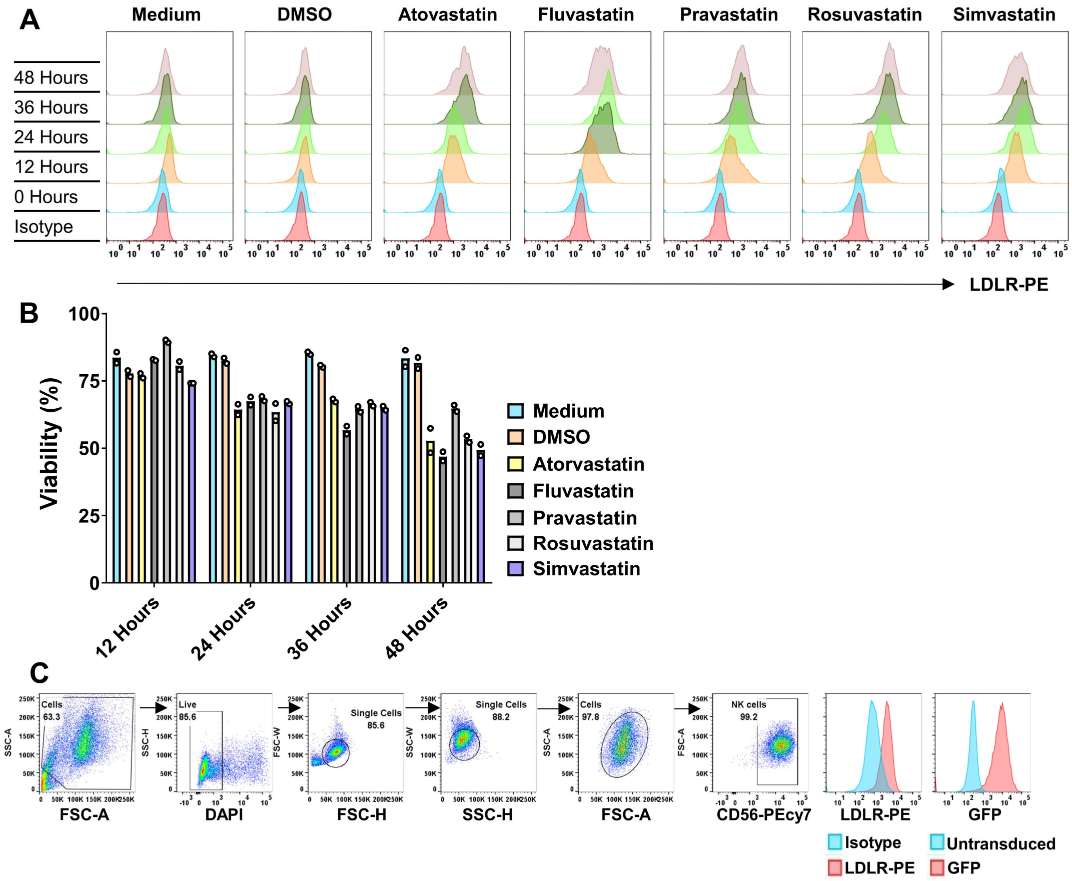

Figure S1 Time course of LDLR expression after statin stimulation.
0.1 x 10^6 cell/mL NK-92 cells were cultured with 5 µM statins for different periods. (A) LDLR expression levels are upregulated by statins in a time-dependent manner Histograms show one representative sample of LDLR expression levels analyzed using flow cytometry. (B) Prolonged culture with statins has a negative impact on NK-92 cell viability. Dead cells were stained with Fixable live/dead Aqua V500. Data shown is one representative of 3 independent assays. (C) Gating strategy of LDLR and GFP expression on NK-92 cells.

A

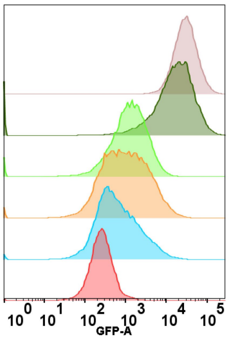

NK92 cells transduced with Protamine Sulfate

MOI	GFP MFI	GFP (%)	Viability (%)
**25**	35334	99,8	75,5
**20**	23655	95,8	72,8
**15**	1837	76,6	74,7
**10**	2123	62	79,5
**5**	1390	47,2	83,4
**Untransduced**	271	4,3	91,6

B

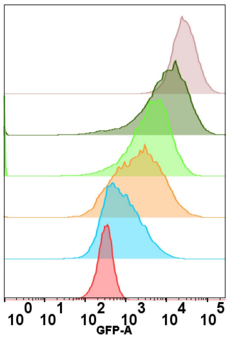

NK92 transduced with Protamine Sulfate + 5µM Rosuvastatin

MOI	GFP MFI	GFP (%)	Viability (%)
**25**	33339	99,7	73,9
**20**	16002	94,6	75,1
**15**	6063	88,9	75,9
**10**	4247	79,8	81,3
**5**	1396	56,9	86,8
**Untransduced**	329	4,93	94,1

C

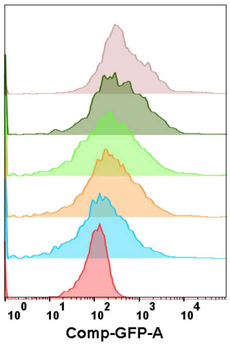

Primary NK transduced with Protamine Sulfate

MOI	GFP MFI	GFP (%)	Viability (%)
**25**	1222	45,60	51,00
**20**	1159	40,70	47,75
**15**	868	27,10	65,50
**10**	627	27,65	74,45
**5**	437	18,20	79,00
**Untransduced**	118	1,12	80,30

D

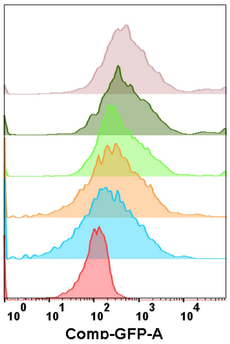

Primary NK transduced with Protamine Sulfate + 5µM Rosuvastatin

MOI	GFP MFI	GFP (%)	Viability (%)
**25**	2580	56,40	46,70
**20**	2024	50,05	50,70
**15**	2120	42,90	62,35
**10**	1014	33,75	77,70
**5**	680	26,75	82,60
**Untransduced**	119	1,35	80,90

Figure S2 Higher virus concentrations promote GFP expression at the expense of cell viability. NK-92 were incubated without (A) or with (B) 5 µM rosuvastatin for 36 hours at 0.1 x 10^6 cells/mL. Different MOI of VSV-G lentivirus were added in the NK-92 cells in the presence of 10 µg/mL protamine sulfate. Histograms and tables show GFP expression (MFI and proportion of positive cells) and cell viability 48 hours after transduction. Similarly GFP expression levels and viability are shown for primary NK cells following lentiviral transduction without (C) or with (D) rosuvastatin pre-incubation.

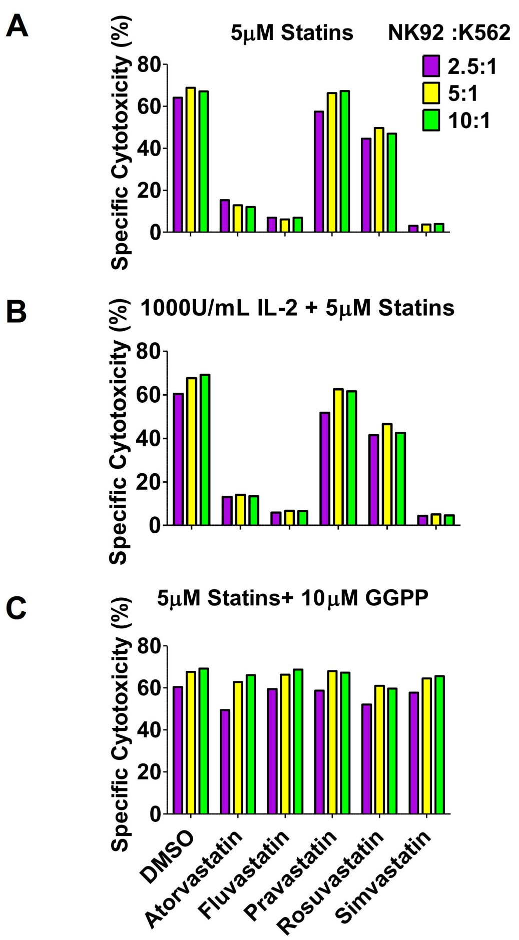

Figure S3 GGPP but not IL-2 can completely reverse the negative impact of statins on the cytotoxic capacity of NK-92 cells.

(A) NK-92 cell cytotoxicity was suppressed by lipophilic statins after 36 hours incubation. However, NK-92 cell cytotoxicity was not changed after pravastatin and not completely be restrained by rosuvastatin. NK-92 cells were seeded at a density of 0.1 X 10^6 cells/mL in 12 well plates for statin stimulation. After removing the supernatant, NK-92 cells were added according to indicated E:T ratio in 96 well-plates. 20,000 K562 cells were seeded per well as target cells. Cytotoxicity assay was performed for 4 hours with different E:T ratios. (B) Statin (5 μM) induced inhibition of NK cell cytotoxicity could not be reversed by 1000 U/mL IL-2. (C) GGPP completely reversed the cytotoxicity suppression of statins on NK-92 cells. Data is shown of one representative assay of 3 independent assays as mean cytotoxicity.

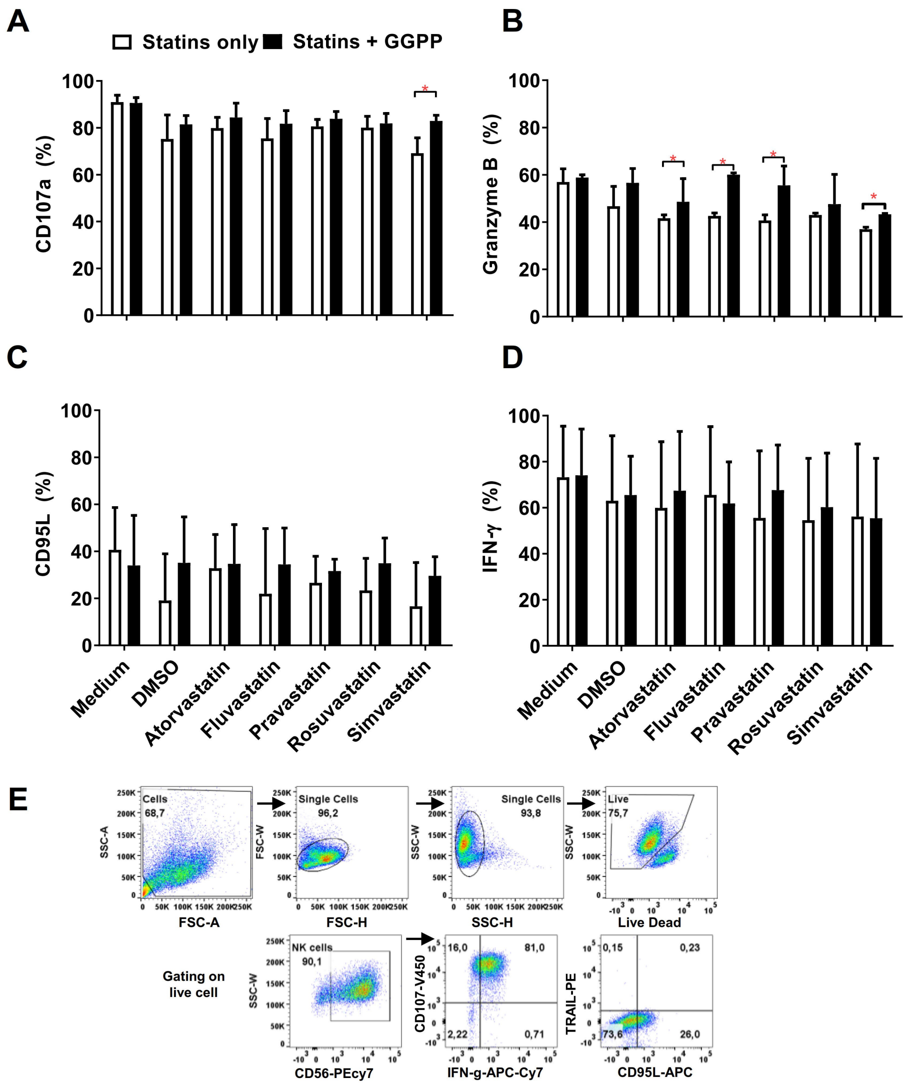

Figure S4 Statins have a negative effect on CD107a and granzyme B expression, which can be reversed using GGPP.

0.1 x 10^6 NK-92 cells were cultured in 1mL with 5 μM statins for 36 hours. Medium was replaced with medium containing CD107a - V450 antibody, and PMA (20 ng/mL) plus ionomycin (1 μg/mL). After 1 hour pre-incubation, 10 μg/mL brefaldin A was added. Cells were stained with surface markers (CD56 and CD95L), and subsequently fixed and permeabilized, followed by IFN-γ intracellular staining. CD107a (A), Granzyme B (B), CD95L (C) and IFN-γ (C) expression in NK-92 were analyzed by flow cytometry. Compared with medium and DMSO, only simvastatin down-regulated CD107a, but was reversed by GGPP. All the statins inhibit the granzyme B, except for rosuvastatin. Fortunately, this negative effect was reversed by adding 10 μM GGPP. (E) Gating strategy of CD107a, CD95L and IFN-γ on NK-92. Data is shown as mean ± SD from N=3 independent experiments performed at different times. Data analysis was performed using a pair signed-rank test between the statins only group and the statins plus GGPP group.

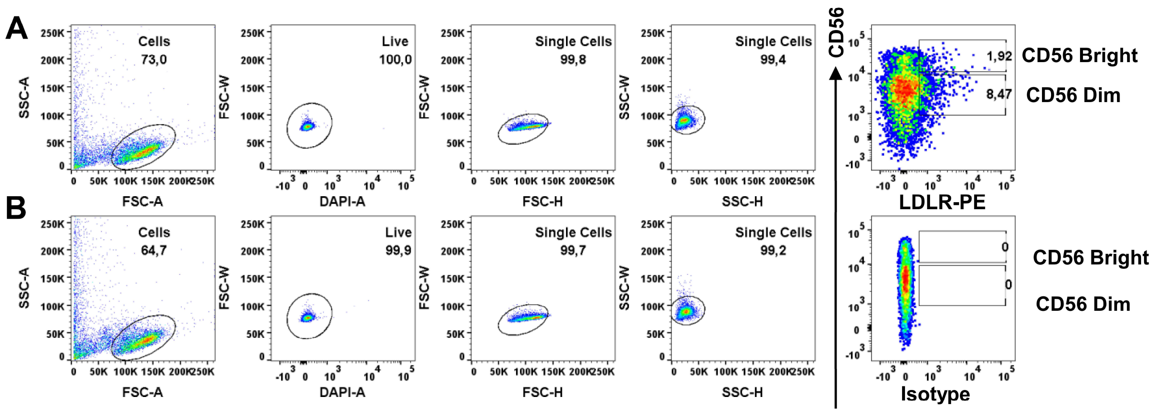

Figure S5 LDLR expression levels on fresh isolated human primary NK cell.
Human NK cells were isolated using negative magnetic selection. LDLR expression levels were analyzed for the CD56 bright and CD56 dim sub-populations using flow cytometry.

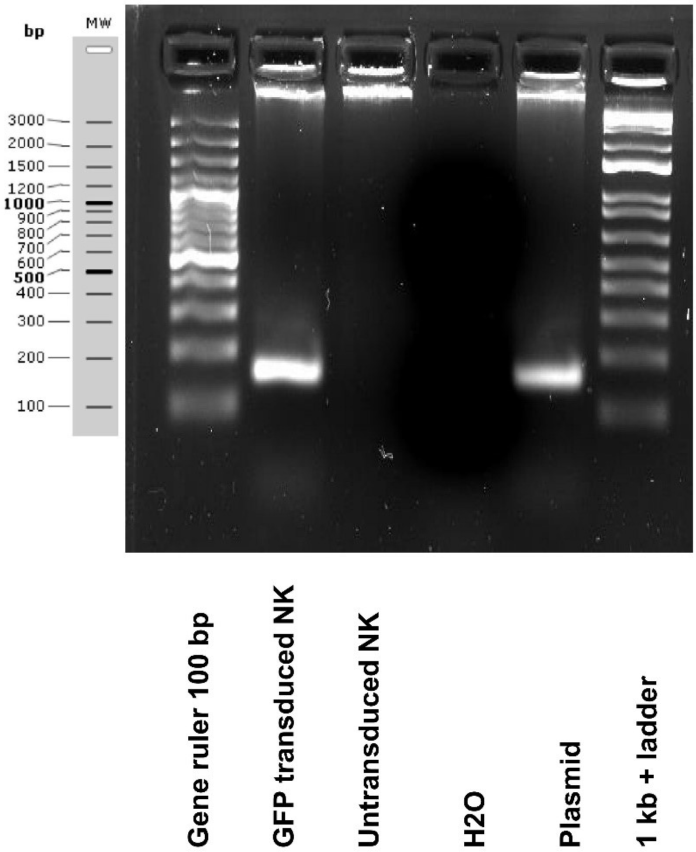

Figure S6 Lentiviral integration in human primary NK cells.
Genomic DNA was extracted 48 hours after VSV-G lentiviral transduction of human primary NK cells. One pair of primer specific amply the GFP fragment with a size at 157 bp. PCR product was running on 1.2 % agarose TAE gel (70 V, 45 minutes). A plasmid encoding GFP was used as positive control, H2O is used as the ‘no template’ control. Untransduced NK cells were used as a negative control.

Supplemental Methods

CD107a degranulation assay

To evaluate NK cell activation by tumor cells or after stimulated with Phorbol 12-myristate 13-acetate (PMA, Sigma Aldrich) plus ionomycin (Sigma Aldrich), CD107a expression on NK cells was analyzed through flow cytometry. NK cells were incubated with PMA (20 ng/mL) plus ionomycin (1 μg/mL) with 2 μL anti-CD107a-Horizon V450 (H4A3, BD Biosciences) in a 96-well plate. After 1 hour of co-culture, 10 μg/mL Brefeldin A (BFA, BD Biosciences) was added. After another 3 hours, the plate was placed on ice to stop the reaction. NK cells were further stained with anti-human CD3 and CD56 antibodies conjugated with appropriate fluorophores surface markers as described above.

Intracellular staining

For intracellular staining of interferon-gamma (IFN-γ), cells were stimulated with PMA (20 ng/mL) plus ionomycin (1 μg/mL) and 10 μg/mL BFA in the culture medium for 4 hours. Then cells were harvested, washed, and stained with fluorochrome-conjugated anti human CD3 and CD56 surface monoclonal antibodies for 30 minutes at 4 °C. Cells were then washed twice and treated with fixation buffer (eBiosciense, Thermo Scientific) for 15 minutes at room temperature. After washing, cells were permeabilized in permeabilization buffer for 30 minutes at 4 °C degree. After washing, fixation and permeabilization, cells were stained with fluorochrome-conjugated anti human IFN-γ antibody (Clone: 45-15, Miltenyi) anti human Granzyme B (Clone REA226, Miltenyi Biotec). After 2 more washes, the cells were analyzed by FACS Canto II (BD Biosciences).

Genomic extraction and PCR

48 hours after transduction, 0.5 x 10^6 primary NK cells were harvested. Primary genomic DNA was isolated using Lucigen QuickExtract™ DNA Extraction Solution (QE0905T, Lucigen Corporation, Wisconsin, USA) as published elsewhere [1]. The GFP construct was amplified using PrimeSTAR HS polymerase kit (R010A, Takara Bio) and the following primers: GFP-forward gtgatgggctacggcttctacc and GFP-reverse primer cctcgtagcggtagctgaag.

Supplemental References

1. Bak, R.O., and Porteus, M.H. (2017). CRISPR-Mediated Integration of Large Gene Cassettes Using AAV Donor Vectors. Cell reports *20*, 750-756.
